# Endocytosis of flavivirus NS1 is required for NS1-mediated endothelial hyperpermeability and is abolished by a single N-glycosylation site mutation

**DOI:** 10.1371/journal.ppat.1007938

**Published:** 2019-07-29

**Authors:** Chunling Wang, Henry Puerta-Guardo, Scott B. Biering, Dustin R. Glasner, Edwina B. Tran, Mark Patana, Trent A. Gomberg, Carmel Malvar, Nicholas T. N. Lo, Diego A. Espinosa, Eva Harris

**Affiliations:** Division of Infectious Diseases and Vaccinology, School of Public Health, University of California, Berkeley, Berkeley, California, United States of America; University of Pennsylvania School of Medicine, UNITED STATES

## Abstract

Arthropod-borne flaviviruses cause life-threatening diseases associated with endothelial hyperpermeability and vascular leak. We recently found that vascular leak can be triggered by dengue virus (DENV) non-structural protein 1 (NS1) via the disruption of the endothelial glycocalyx-like layer (EGL). However, the molecular determinants of NS1 required to trigger EGL disruption and the cellular pathway(s) involved remain unknown. Here we report that mutation of a single glycosylated residue of NS1 (N207Q) abolishes the ability of NS1 to trigger EGL disruption and induce endothelial hyperpermeability. Intriguingly, while this mutant bound to the surface of endothelial cells comparably to wild-type NS1, it was no longer internalized, suggesting that NS1 binding and internalization are distinct steps. Using endocytic pathway inhibitors and gene-specific siRNAs, we determined that NS1 was endocytosed into endothelial cells in a dynamin- and clathrin-dependent manner, which was required to trigger endothelial dysfunction *in vitro* and vascular leak *in vivo*. Finally, we found that the N207 glycosylation site is highly conserved among flaviviruses and is also essential for West Nile and Zika virus NS1 to trigger endothelial hyperpermeability via clathrin-mediated endocytosis. These data provide critical mechanistic insight into flavivirus NS1-induced pathogenesis, presenting novel therapeutic and vaccine targets for flaviviral diseases.

## Author summary

Vascular leak is a hallmark of severe dengue disease. Recently, our group revealed a critical role for NS1 in induction of endothelial hyperpermeability and vascular leakage in an endothelial cell-intrinsic manner. However, the upstream pathway triggered by NS1, as well as the molecular determinants of NS1 required for this phenomenon, remain obscure. Gaining insight into this endothelial cell-intrinsic pathway is critical for understanding dengue pathogenesis, developing novel antiviral therapies, and developing NS1-based vaccine approaches that pose a minimal risk of antibody-dependent enhancement. Our current study expands our knowledge of this novel pathway not only by identifying the requirement of internalization of secreted NS1 via clathrin-mediated endocytosis, but also by pinpointing the NS1 molecular determinant (N207) required to trigger vascular leak. Further, our work identifies N207 as a residue conserved among multiple flaviviruses (Zika virus and West Nile virus, in addition to DENV), which is critical for NS1-mediated vascular leak in biologically relevant human endothelial cells modeling interstitial compartments in the lung or the blood-brain barrier. Thus, our study identifies endocytosis and a single amino acid (N207) of the NS1 viral toxin as critical for pan-flavivirus pathogenesis, representing a novel target for anti-flaviviral therapy and vaccine development.

## Introduction

Dengue virus (DENV) is a mosquito-borne flavivirus, and infection with any of its four serotypes (DENV1-4) can result in inapparent infection, classic dengue fever, or dengue hemorrhagic fever/dengue shock syndrome–severe manifestations characterized by vascular leak that can lead to shock and death [1]. West Nile virus (WNV) is a related flavivirus that causes encephalitis [2], and Zika virus (ZIKV), which recently emerged and generated large epidemics across the Americas, can cause Guillain-Barré syndrome in adults and microcephaly and other congenital birth defects in babies born to women infected during pregnancy [3–7]. The 10.7-kb RNA genome of flaviviruses encodes three structural and seven non-structural (NS) proteins, including NS1. We recently described a novel cell-intrinsic role for DENV NS1 in increasing permeability of human endothelial cell monolayers *in vitro* and systemic vascular leak *in vivo* via disruption of components of the endothelial glycocalyx-like layer (EGL) [8–10]. This EGL pathway is distinct from the cytokine-mediated pathway involving activation of peripheral blood mononuclear cells (PBMCs) previously described [10, 11]. The NS1-mediated degradation of the EGL involves activation of the protease cathepsin L, heparanase, and sialidases, which in turn disrupt the EGL. However, the upstream pathway initiated by NS1, resulting in cathepsin L activation, EGL degradation, and therefore vascular leak, as well as the molecular determinants of NS1 required for inducing endothelial hyperpermeability, have not yet been identified.

All flavivirus NS1 genes are highly homologous and encode a 352-amino acid polypeptide with slight variation in glycosylation status that results in a molecular weight of 46–55 kDa [12]. High-resolution structures of the C-terminal half [13] and full-length [14] DENV NS1, as well as the closely related WNV NS1, revealed dimers containing distinct domains for membrane association and interactions with the immune system and provided structural insights into flavivirus NS1 assembly and antibody recognition [14]. In flavivirus-infected cells, NS1 dimers are found intracellularly and also on the cell surface, while secreted forms are comprised of atypical barrel-shaped hexamers containing lipid cargo [15].

DENV NS1 contains two conserved N-linked glycans (N-glycans) at Asn-130 (N130) and Asn-207 (N207), and NS1 proteins derived from different cells/species exhibit distinct types of N-glycosylation [16]. Previous studies investigating the importance of the N-glycans on NS1 found that DENV2 deglycosylated at either site exhibited significant attenuation of neurovirulence in mice compared to the wild-type (WT) virus [17]. However, the mechanism of this attenuated virulence has never been elucidated, due in part to the fact that NS1 N-glycosylation mutant viruses are unstable, with significant defects in viral replication and proliferation that prevent further conclusive mechanistic study [17]. This has been a major roadblock in efforts to study the dual role of NS1 in viral replication and pathogenesis. In mammalian cell-derived DENV NS1, complex glycans are attached at Asn-130 and high-mannose glycans are attached at Asn-207 [18]; these N-glycans are involved in NS1 dimer stability and secretion [18, 19].

In this study, to investigate the role of these N-glycosylation sites directly on NS1-induced vascular leak independently from their roles in viral replication, we generated recombinant NS1 in a mammalian expression system, which incorporates human glycosylation patterns. We sought to investigate whether the glycosylation status of NS1 may contribute to its ability to induce endothelial hyperpermeability. Using both transendothelial electrical resistance (TEER) and an immunofluorescence assay (IFA) with confocal microscopy, we show that the NS1-N207Q mutant does not induce endothelial barrier dysfunction or degradation of the EGL of human pulmonary microvascular endothelial cells (HPMEC). Further, we demonstrate that there are no differences in cell binding *per se* between DENV WT NS1 and the NS1-N207Q mutant, but the latter is not efficiently internalized by endothelial cells, indicating that internalization of NS1 is required for endothelial cell-intrinsic pathogenesis. Furthermore, we show that endothelial cells internalize NS1 via clathrin-mediated endocytosis and that its inhibition by either small molecule inhibitors or siRNA knock-down prevents NS1-induced hyperpermeability and EGL degradation *in vitro* and vascular leak *in vivo*. Finally, we identify N207 as a conserved residue of NS1 among multiple flaviviruses (such as DENV, ZIKV, and WNV) that is critical for NS1 internalization and endothelial hyperpermeability in biologically relevant human cells. Taken together, our results indicate that the N207 glycosylation site is required for NS1 internalization by endothelial cells and that clathrin-mediated endocytosis is a key step in the pathway leading to flavivirus NS1-induced endothelial hyperpermeability and barrier dysfunction.

## Results

### DENV NS1-N207Q mutant protein does not induce endothelial hyperpermeability in human endothelial cells

**Fig 1:**
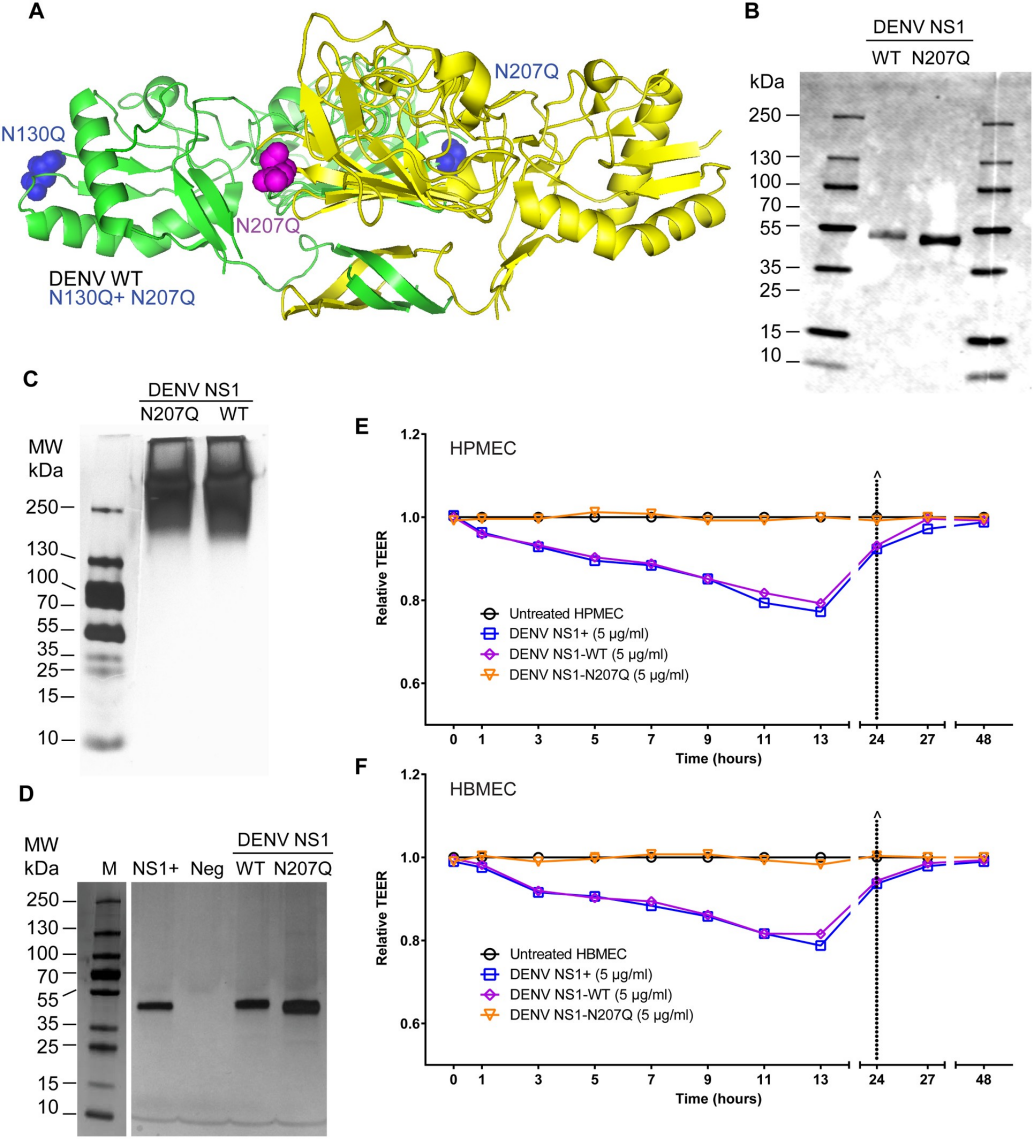
Mutation of the N-glycosylation site 207 prevents DENV NS1-induced hyperpermeability of endothelial cells. **(A)** Structural model of a DENV2 NS1 dimer showing site-specific mutations. Color-coding highlights two NS1 monomers (one in green, one in yellow) and amino acid changes in corresponding constructs (blue highlights changes in the green monomer; pink highlights changes in the yellow monomer). WT, N130Q, N207Q, and N130Q+N207Q are all on the DENV2 NS1 backbone. PDB ID: 4O6B [14]. (**B)** Western blot of SDS-PAGE of in-house purified and dialyzed DENV NS1-WT and NS1-N207Q mutant, showing monomeric form, using anti-NS1 mAb (7E11) for detection. **(C)** Silver stain of native PAGE showing oligomeric forms of both purified and dialyzed in-house DENV NS1-WT and NS1-N207Q mutant (MW: >250 kDa). **(D)** Silver stain of SDS-PAGE gels of purified and dialyzed in-house DENV NS1-WT and NS1-N207Q NS1 mutant, showing purity, using the Pierce Silver Stain Kit. Positive control, NS1^+^, DENV NS1 from Native Antigen Company; Neg, dialyzed elution buffer. Molecular weight marker, 10-250-kDa protein ladder. **(E-F)** DENV NS1 triggers endothelial permeability of HPMEC (**E**) and HBMEC (**F**) monolayers evaluated by TEER (Ohms) at the indicated time-points. Relative TEER was calculated as previously described [8]. Data are representative of 2–3 individual experiments. NS1+, NS1 from Native Antigen Company.

To examine the role of NS1 glycosylation status on endothelial barrier dysfunction, we generated several NS1 constructs targeting glycosylation sites in DENV2 NS1 (henceforth referred to as DENV NS1), including WT, the single N-glycosylation mutants N130Q and N207Q, and the double mutant N130Q+N207Q (Fig 1A and S1 Table). DENV NS1-WT and DENV NS1-N207Q were successfully secreted and purified as oligomers (MW: >250 kDa) (Fig 1B and 1C and S1A Fig); the DENV NS1-N207Q mutant (monomer ~43 kDa) was slightly smaller than the NS1-WT monomer (~48 kDa) due to loss of the N207 glycosylation site (Fig 1B), consistent with previous publications [19]. In contrast, the N130Q mutant was not efficiently secreted, nor was the double mutant N130Q+N207Q (S1B Fig); thus, these two constructs were not further characterized. 6xHis-tagged recombinant DENV NS1 proteins were purified using nickel-nitrilotriacetic acid resin agarose beads and subjected to dialysis; the resulting purified DENV NS1 proteins (WT and N207Q) were visualized by silver stain and found to exhibit purity similar to commercially available NS1 (>95%), as previously described [8] (Fig 1D). To confirm the successful removal of an N-glycan at position 207, we digested WT NS1 (both commercial and in-house produced) and the NS1-N207Q mutant with endoglycosidase H (Endo H; removes high mannose glycans) or PNGase F (removes complex glycans and high mannose glycans), and measured their digestion sensitivity through observation of a gel shift by Western blot. The commercial and in-house produced WT NS1 proteins were sensitive to digestion by both Endo H and PNGase F, suggesting they possessed both the high mannose glycan at position 207 as well as the complex glycan at position 130. In contrast, the NS1-N207Q mutant was sensitive to digestion with PNGase F but not Endo H, suggesting that it retained the complex glycan at position 130 but no longer possessed the high mannose N-glycan at position 207, confirming its removal by the N207Q mutation (S1C Fig).

We next compared the size and stability of the NS1-WT and NS1-N207Q proteins. When subjected to size-exclusion chromatography, both our in-house produced NS1-WT and NS1-N207Q eluted at similar fractions, suggesting they are of comparable size and conformation (S2A and S2B Fig). We then utilized an NS1-specific monoclonal antibody (9NS1) previously reported to detect NS1 in a native but not in a denatured conformation [20], to further confirm that NS1-WT and NS1-N207Q were in a comparable conformation. Indeed, we found that 9NS1 recognized WT NS1 (both commercial and in-house produced) comparably to NS1-N207Q in native conditions but did not detect any of the three under denaturing conditions. This confirms both that 9NS1 functions as a conformational antibody and that the conformation of NS1-WT and NS1-N207Q is comparable (S3A Fig). As expected, several additional NS1-specific monoclonal antibodies that are not conformation-dependent, as well as an antibody targeting the 6xHis-tag, recognized all NS1 proteins comparably in native and denaturing conditions (S3A Fig). Next, to confirm that our purified and dialyzed NS1 proteins were stable at 37°C over the time-frame relevant for our *in vitro* cell culture-based assays, we diluted the NS1 proteins in our standard cell culture medium and incubated these proteins at 37°C for varying times. We found that both NS1-WT and NS1-N207Q are stable under these conditions both at time-points relevant for our *in vitro* assays (7 hours) and even at longer time-points (48 hours) (S3B–S3D Fig). As expected, in these experiments, the addition of SDS or proteinase K degraded NS1 (S3B–S3D Fig). Taken together, these data indicate that our purified NS1 proteins (both NS1-WT and NS1-N207Q) are comparable in size, conformation, and stability.

To investigate the functional role of NS1 glycosylation on NS1-mediated pathogenesis, we first studied the effects of the proteins on endothelial barrier function by measuring the TEER of HPMEC, since a major site of vascular leak in severe dengue occurs in the lungs [8]. Our DENV NS1-WT triggered a similar decrease in TEER values as commercial DENV2 NS1 (DENV NS1+) (Fig 1E), indicating disruption of endothelial barrier integrity. In contrast, the relative TEER of the DENV NS1-N207Q mutant was similar to that of mock-treated cells, demonstrating that mutation of the N207 glycosylation site in DENV NS1 completely prevents induction of endothelial hyperpermeability in HPMEC (Fig 1E and S4A Fig). Similar results were observed in human brain microvascular endothelial cells (HBMEC) (Fig 1F and S4B Fig); these cells model barrier function of the blood-brain barrier, which is relevant due to cases of dengue encephalitis and reports of DENV detected in the brain in autopsies of dengue shock cases [21, 22]. Together, these data suggest that the N207 glycosylation site is a critical molecular determinant of DENV NS1-mediated pathogenesis.

### DENV NS1-N207Q mutant protein binds to cells but does not trigger degradation of the endothelial glycocalyx-like layer

**Fig 2:**
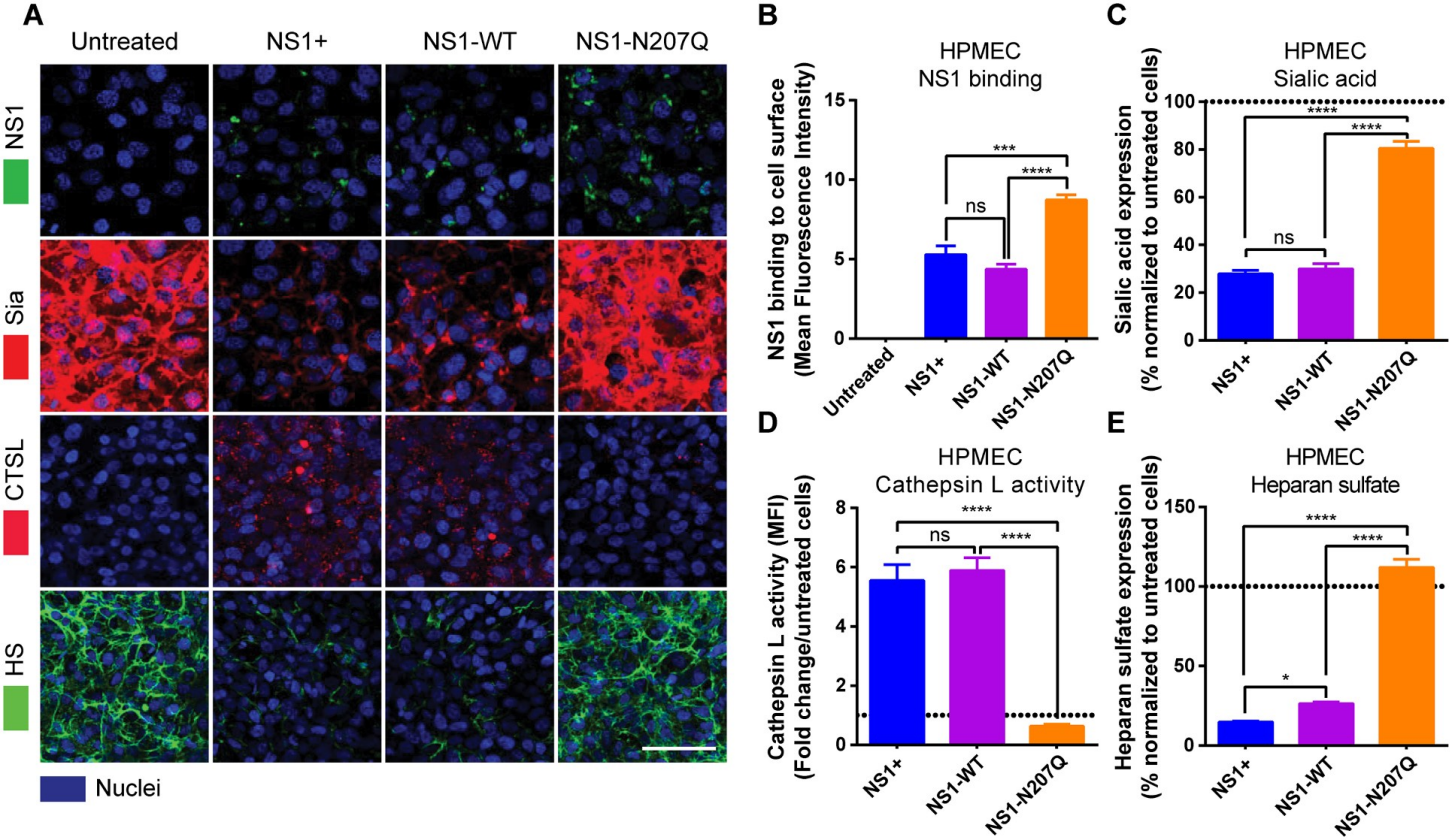
Mutation of the N-glycosylation site 207 prevents DENV NS1-induced cathepsin L and sialidase activation and EGL disruption on endothelial cells. **(A)** NS1 protein binding (green, top row) to HPMEC 6 hpt at 37°C was visualized via IFA. The integrity of the EGL was assessed by the presence of sialic acid (Sia) surface expression, stained with WGA-A647 (red, 2^nd^ row; merge of NS1 and Sia), as well as cathepsin L (CTSL) activity (red, 3^rd^ row) and heparan sulfate [9] surface expression (green, bottom row), in HPMEC 6 hpt with NS1 at 37°C, as visualized via IFA. Nuclei were stained with Hoechst (blue). Images (20X; scale bars, 50 μm) are representative of 2 independent experiments. **(B)** Mean Fluorescence Intensity (MFI) was used to quantify the amount of NS1 binding to the cell surface, **(C)** sialic acid surface expression, **(D)** cathepsin L activity, and **(E)** heparan sulfate expression on HPMEC in **Fig 2A**. The means ± standard error of the mean (SEM) of two individual experiments run in duplicate are shown. ns = not significant; *, p<0.05; ***, p<0.001; ****, p<0.0001.

We previously demonstrated that DENV NS1 binds to endothelial cells and activates cathepsin L, which then activates heparanase, leading to cleavage of heparan sulfate from proteoglycans on the cell surface [9]. NS1 also activates sialidases, which cleave sialic acid on the cell surface [9]. Together, these events contribute to NS1-induced degradation of the EGL, leading to endothelial barrier dysfunction [9, 10]. To mechanistically determine why the DENV NS1-N207Q mutant no longer triggers hyperpermeability of HPMEC, we first asked whether the DENV NS1-N207Q mutant was still able to bind to the surface of endothelial cells. We found that both the DENV NS1-N207Q mutant and WT DENV NS1 (both commercial and in-house-produced) bound to the surface of HPMEC as visualized by IFA (Fig 2A and 2B and S5A and S5B Fig). However, the DENV NS1-N207Q mutant did not trigger EGL disruption, as determined by disruption of sialic acid, activation of cathepsin L, and cleavage of heparan sulfate, visualized by IFA at 1 and 6 hours post-treatment (hpt) with NS1 (Fig 2A–2E and S5A and S5C Fig). These results suggest that the N-glycosylation of DENV NS1 at residue 207 plays a role in the activation of the endothelial cell-intrinsic mechanisms leading to disruption of the EGL.

### DENV NS1-N207Q mutant is not internalized by endothelial cells

**Fig 3:**
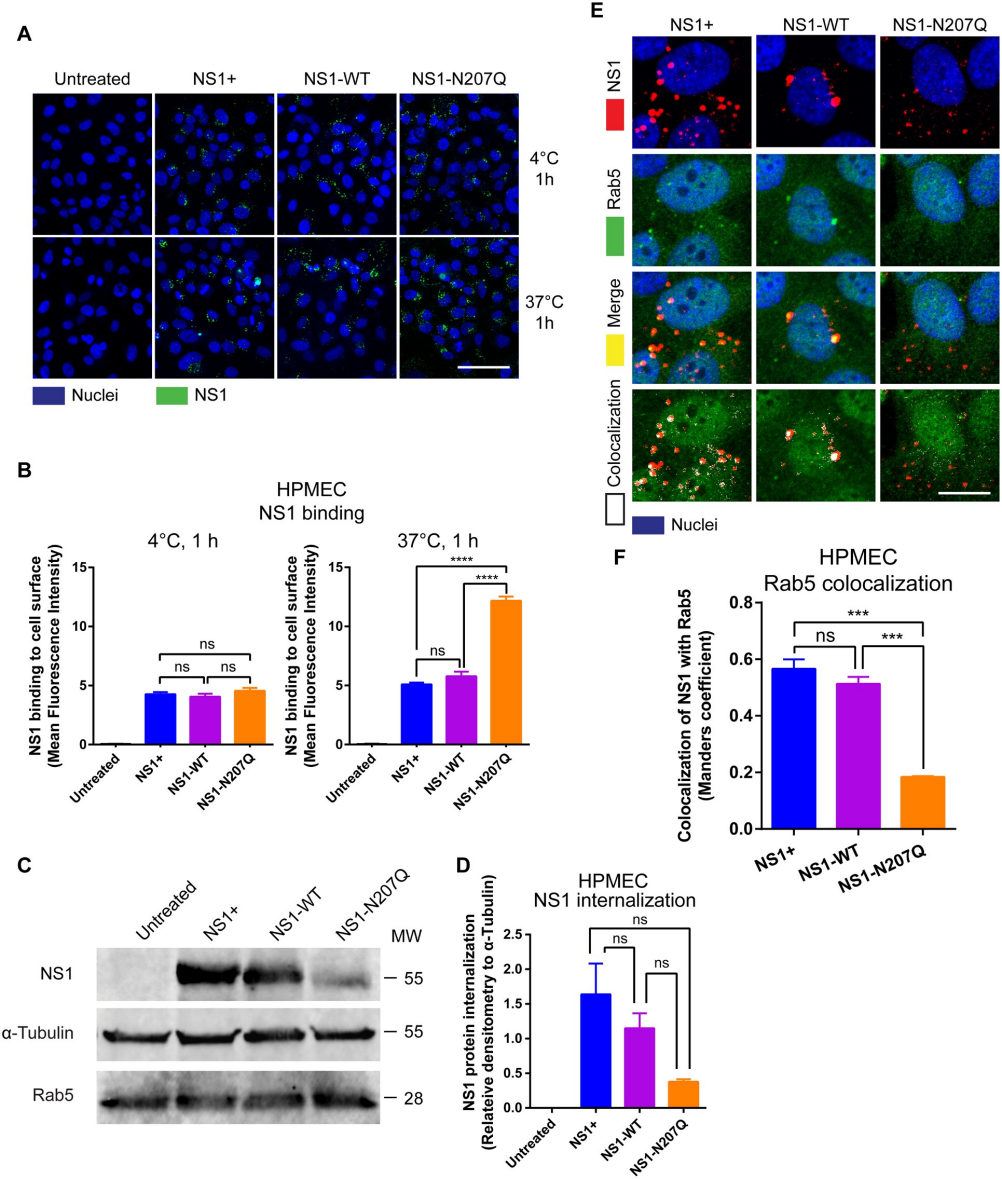
Mutation of the N-glycosylation site 207 of DENV NS1 results in defective NS1 internalization. **(A)** NS1 protein binding (green) to HPMEC 1 hpt at 4°C (top row) or 37°C (bottom row) was visualized via IFA. Images (20X; scale bars, 50 μm) are representative of 2 independent experiments. **(B)** MFI was used to quantify the amount of NS1 binding to the cell surface 1 hpt at 4°C and 37°C in **Fig 3A**. The means ± SEM of two individual experiments run in duplicate are shown. ns = not significant; ****, p<0.0001. **(C)** Confluent HPMEC monolayers were exposed to 10 μg/ml of different NS1 proteins and incubated at 37°C for 1 hours. Trypsin was used to remove surface-bound NS1, and cell lysates were analyzed by Western blot. Western blot shows detection of the internalized DENV NS1 protein, GAPDH (loading control), and Rab5, an early endosome marker. (**D**) Quantitation of C. Each bar represents the mean ± standard error of the mean (SEM) of densitometry values normalized to the control protein α-tubulin from two independent experiments. **(E)** Co-localization of NS1 proteins (red), as indicated, with Rab5 (green) in HPMEC. Co-localization is shown in yellow in merge image or white in co-localization panel (JACoP, ImageJ). Nuclei are stained with Hoechst (blue). Images (40X; scale bars, 10 μm) are representative of 2 individual experiments run in duplicate. NS1+, NS1 from Native Antigen Company. **(F)** Quantification of DENV NS1 and Rab5 colocalization in HPMEC in **Fig 3E**. Quantification of the amount of spatial overlap between the two signals (NS1 in red and Rab5 in green) in **Fig 3E** was obtained using four different frames from the maximum projections of two RGB images based on the object-based approach (JACoP) and defined by the Manders’ Coefficient as previously described [59]. The colocalization coefficient was normalized taking into account the signal obtained from 300 cells per image. Each bar represents the mean ± SEM of MFI values in the colocalization analyses obtained from two independent experiments. NS1+, NS1 from Native Antigen Company. ns = not significant; ***, p<0.001.

Though the DENV NS1-N207Q mutant was still able to bind to the surface of endothelial cells without leading to disruption of the EGL, it surprisingly appeared to bind to the surface of HPMEC at substantially higher levels than WT DENV NS1 (both commercial and in-house-produced) after 6 hours at 37°C (Fig 2A and 2B). To further investigate this finding, we evaluated NS1 levels on the surface of HPMEC at 4°C, a temperature at which internalization via endocytosis should be greatly reduced [23, 24]. This examines whether the apparent enhanced binding of DENV NS1-N207Q at 37°C was due to an intrinsically enhanced ability of the DENV NS1-N207Q mutant to bind to endothelial cells or through some other mechanism, such as deficient endocytosis. We found that the DENV NS1-N207Q mutant bound to HPMEC at similar levels as both commercial and in-house-produced WT DENV NS1 when incubated at 4°C for 1 hour, suggesting the DENV NS1-N207Q does not intrinsically bind more to HPMEC compared to WT DENV NS1 (Fig 3A, top row, and Fig 3B, left panel). Instead, when NS1 proteins were incubated with HPMEC at 37°C (allowing for protein internalization) for 1 hour, the DENV NS1-N207Q mutant again bound at higher levels compared to WT NS1, suggesting that it remained on the cell surface, while both commercial and in-house-produced WT NS1 proteins were internalized (Fig 3A, bottom row, and Fig 3B, right panel).

NS1 is known to bind to heparan sulfate moieties on the surface of endothelial cells [25]. To determine whether the NS1-N207Q mutant still required heparan sulfate moieties to bind to the surface of endothelial cells, we pretreated cells with recombinant heparanase to selectively remove heparan sulfate from the cell surface, and then compared the ability of NS1-WT and the NS1-N207Q mutant to bind to these cells. As expected, the in-house produced NS1-WT exhibited a significant decrease in binding to heparanase-treated cells compared to untreated cells. Similarly, the NS1-N207Q mutant also exhibited a significant decrease in binding to heparanase-treated cells compared to the untreated cells, suggesting that the NS1-N207Q mutant still requires heparan sulfate to bind to the endothelial cell surface (S6A and S6B Fig). As expected, levels of heparan sulfate were dramatically decreased in endothelial cells pretreated with heparanase compared to untreated control cells (S6C Fig).

Our previous observations, and the observations of others, support the concept of NS1 internalization by endothelial cells [9, 26]. To further support the notion that the DENV NS1-N207Q mutant has a defect in internalization, we incubated WT DENV NS1 and DENV NS1-N207Q with HPMEC at 37°C and compared levels of NS1 after stripping surface-bound NS1 from HPMEC; the remaining NS1 is presumed to have been internalized. We found that WT DENV NS1 (both commercial and in-house-produced) appeared at higher levels than the DENV NS1-N207Q mutant in the HPMEC cell lysate at 1 hpt following trypsin-mediated removal of surface-bound NS1, as measured by Western blot, supporting our hypothesis that the DENV NS1-N207Q mutant has an internalization defect (Fig 3C and 3D). Non-trypsin-treated cells served as a control for the initial levels of NS1 added to cells (S7A Fig). To visualize intracellular WT NS1, we incubated NS1 with HPMEC at 37°C for 1 hour and examined permeabilized cells by IFA for colocalization of WT DENV NS1-WT with Rab5, a regulatory GTPase associated with early endosomes [27]. Both WT NS1 and NS1-N207Q proteins were detected; however, only WT DENV NS1, but not DENV NS1-N207Q, colocalized with Rab5 (Fig 3E and 3F and S7B Fig). This pattern can also be visualized in an animated single 2D projection of NS1 internalization in HPMEC created using multiple confocal Z-stack images (S1 and S2 Movies). After binding to the cell surface, the WT NS1 protein (in *red*, indicated by white arrowheads) moves inside the cell, where it encounters Rab5 (in *green*), which results in a coalescence signal (in *yellow*), suggesting their spatial colocalization in the cell. Interestingly, as the Z stack images move into a deeper Z axial space, this colocalizing signal disappears, and only the red signal for NS1 persists, suggesting its mobilization into a different intracellular compartment (S1 Movie). This process does not occur with the NS1-N207Q mutant protein, where most of the NS1 (in *red*) seems to remain on the cell surface, as little to no signal appears to colocalize with Rab5, suggesting a lack of internalized protein (S2 Movie). To further demonstrate the localization of NS1 in the early endosome, we show that NS1 also colocalizes with overexpressed Rab5-GFP as well as an additional early endosome marker (EEA1) (S7C and S7D Fig) Collectively, these data demonstrate that while both WT DENV NS1 and DENV NS1-N207Q bind comparably to endothelial cells, only WT DENV NS1 is internalized, suggesting that endothelial cell binding and internalization may be distinct stages of NS1-induced cell-intrinsic endothelial hyperpermeability. Based on these results, we hypothesized that DENV NS1 internalization by endothelial cells is a critical step in the activation of EGL disruption and endothelial hyperpermeability.

### DENV NS1 is internalized via clathrin-mediated endocytosis, which is required to trigger endothelial hyperpermeability, EGL degradation, and vascular leak

**Fig 4:**
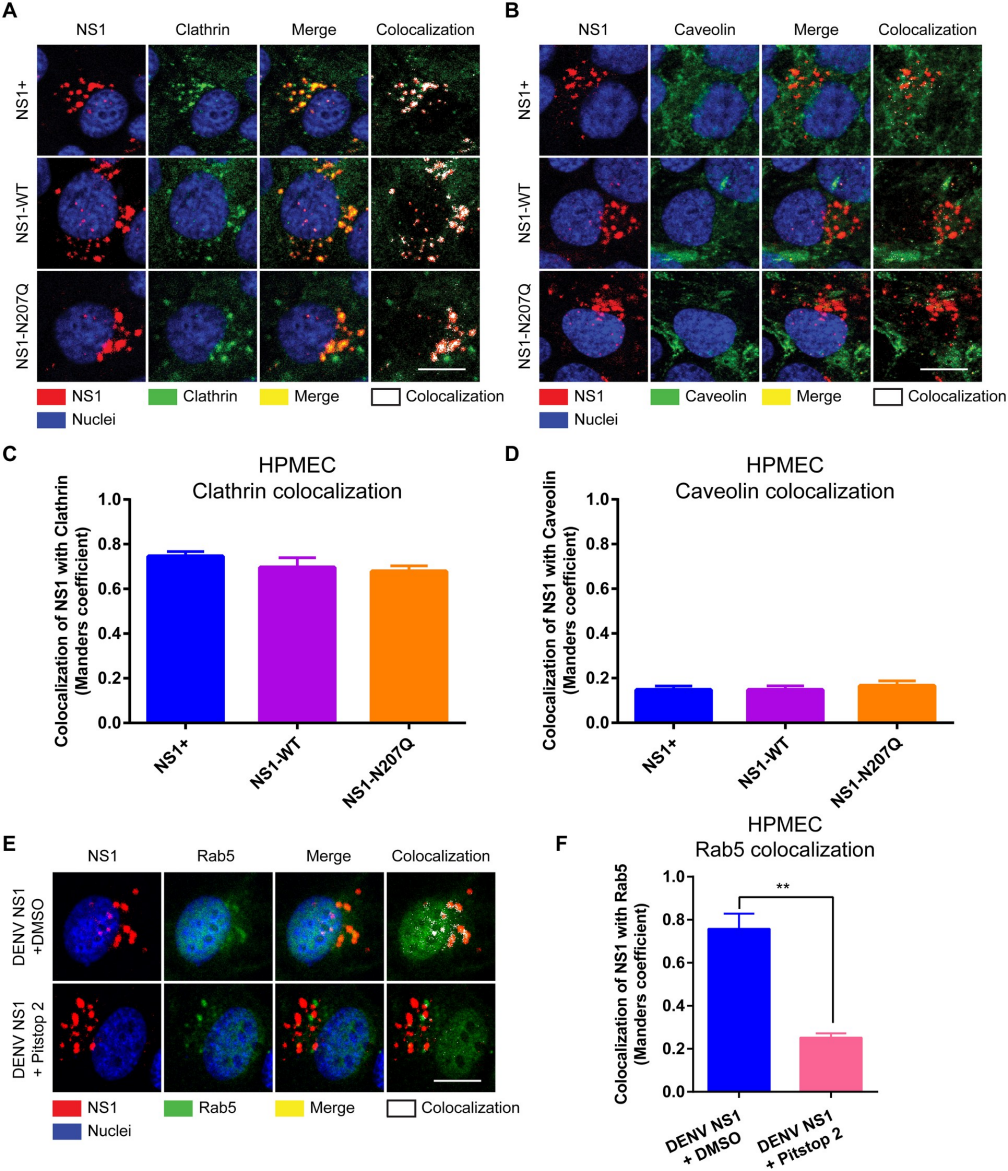
DENV NS1 colocalizes with clathrin but not caveolin on the surface of HPMEC, and clathrin-mediated endocytosis inhibitors prevent colocalization of NS1 and Rab5. Confluent HPMEC monolayers were exposed to 10 μg/ml of different NS1 proteins and incubated at 37°C for 30 minutes. Colocalization of NS1 proteins (red, left column), as indicated, with either **(A)** clathrin or **(B)** caveolin (green, second column) in HPMEC. Colocalization is shown in yellow in merge images (third column) or white in colocalization panels (fourth column; JACoP, ImageJ). Nuclei were stained with Hoechst (blue). Images (40X; scale bars, 5 μm) are representative of 2 independent experiments performed in duplicate. **(C-D)** Quantification of the amount of spatial overlap between the two signals, NS1 and clathrin **(C)**, or NS1 and caveolin **(D)** in **Fig 4A** and **4B**, respectively. The means ± SEM of two individual experiments run in duplicate are shown. **(E)** Clathrin-mediated endocytosis inhibitor Pitstop 2 prevents colocalization of WT DENV NS1 with Rab5 in HPMEC. Colocalization of NS1 proteins (red, first column) on HPMEC treated with either DMSO (top row) or Pitstop 2 (bottom row) with Rab5 (green, second column) 1.5 hpt with NS1. Colocalization is shown in yellow in merge images (third column) or white in colocalization panels (fourth column; JACoP, ImageJ). Nuclei were stained with Hoechst (blue). Images (40X; scale bars, 5 μm) are representative of 2 independent experiments performed in duplicate. **(F)** Quantification of the amount of spatial overlap between the two signals, NS1 and Rab5 in **Fig 4E**. The means ± SEM of two individual experiments run in duplicate are shown. ns = not significant; **, p<0.01.

Endocytosis is a major route for transporting molecules into cells to regulate many cellular signaling processes and is commonly facilitated by two main endocytic molecules: clathrin and caveolin [28]. To interrogate the mechanism by which DENV NS1 is internalized by human endothelial cells, we incubated DENV NS1 with HPMEC and examined localization of internalized NS1 with clathrin and caveolin. We detected colocalization of WT DENV NS1 (both commercial and in-house-produced) with clathrin (Fig 4A and 4C) but not with caveolin in both HPMEC (Fig 4B and 4D) and HBMEC (S8A–S8D Fig), suggesting that NS1 is internalized into endothelial cells in a clathrin-dependent manner. Interestingly, although DENV NS1-N207Q colocalized with clathrin comparably to DENV WT NS1 in HPMEC (Fig 4A and 4C), only WT DENV NS1 colocalized with the early endosome marker Rab5 (Fig 3E–3F). These data suggest that while binding of the DENV NS1-N207Q mutant to endothelial cells is sufficient to recruit clathrin to the NS1 binding site, it is not sufficient to promote internalization [29, 30].

To determine whether internalization of DENV NS1 is dependent on clathrin, we utilized a common chemical inhibitor of clathrin-mediated endocytosis. Pitstop 2 is a selective inhibitor of clathrin-mediated endocytosis that acts by blocking ligand access to the clathrin N-terminal domain [31]. Pretreating HPMEC with Pitstop 2 abrogated NS1 colocalization with Rab5, suggesting inhibition of internalization (Fig 4E and 4F). These data suggest that DENV NS1 is internalized in a clathrin-dependent manner.

Cathepsin L and downstream cellular enzymes are activated in endothelial cells treated with NS1 [9]; as such, we hypothesized that internalized NS1 would colocalize with cathepsin L. Since the peak of NS1-induced cathepsin L activation occurs as early as 30 minutes post-treatment [9], we conducted an internalization time-course experiment to monitor the lifespan of NS1 within cells to determine whether internalization kinetics correlate with cathepsin L activation. WT NS1 (DENV NS1+) was incubated with HPMEC for 45 minutes at 4°C to allow NS1 to bind to cells. Afterwards, cells were washed with cold PBS to remove any unbound NS1 and were then allowed to incubate at 37°C for varying times. Upon temperature shift to 37°C, NS1 accumulated rapidly in HPMEC, reaching a maximum at 15 minutes post-temperature shift, and then rapidly decreased until dropping to near undetectable levels at 3 hours post-temperature shift (S9A–S9C Fig). These kinetics correlate well with the previously observed kinetics of NS1-triggered activation of cathepsin L [9]. Intriguingly, NS1 colocalized with the early endosome marker Rab5, the lysosomal marker LAMP1, and cathepsin L, suggesting that the presence of NS1 in these intracellular compartments may activate cathepsin L (Fig 3E, S9D and S9E Fig). These data indicate that NS1 rapidly and transiently internalizes into cells and localizes to compartments where cathepsin L is present, suggesting that internalization of NS1 may be required for activation of cathepsin L, and thus downstream disruption of the EGL.

**Fig 5:**
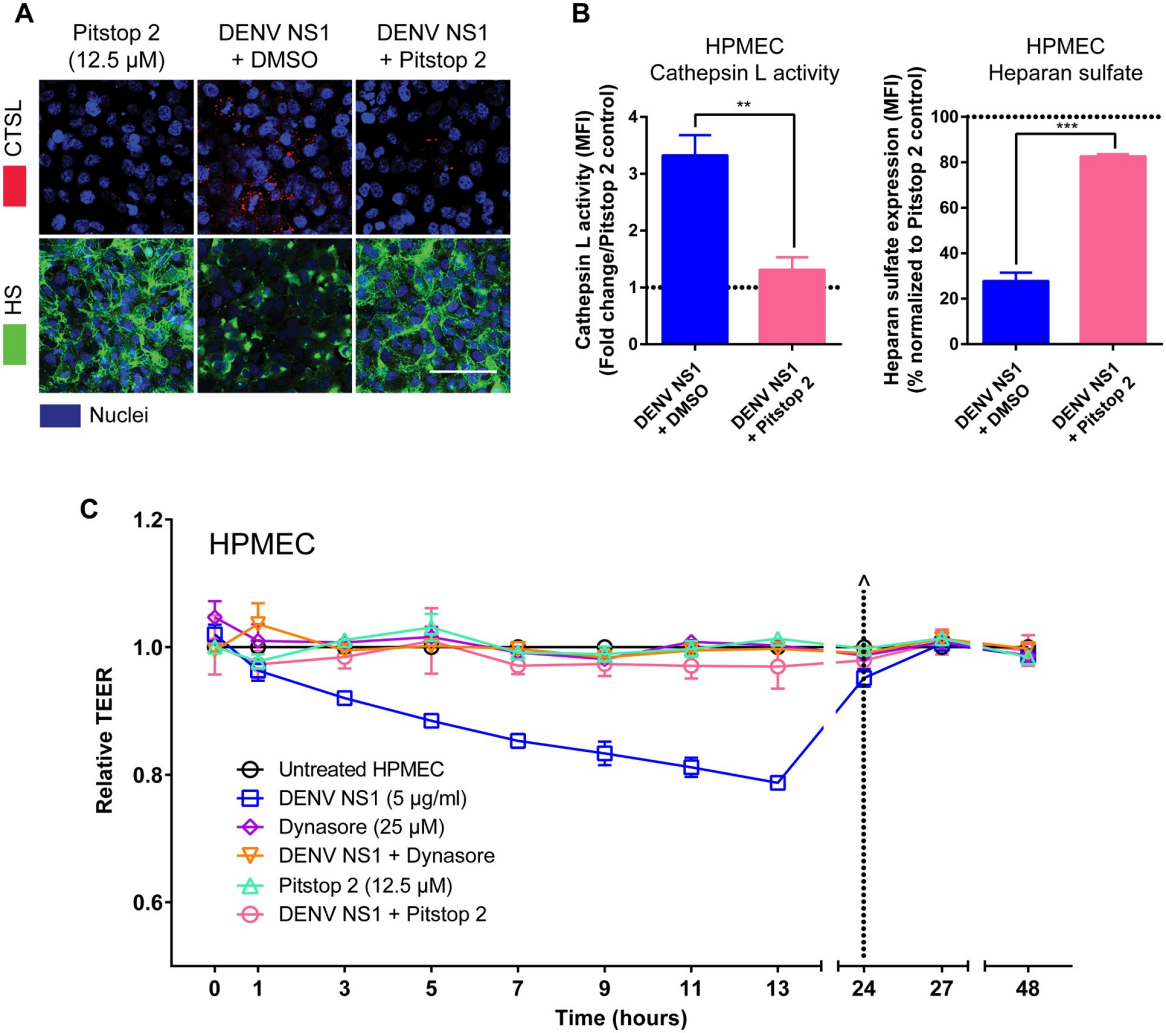
Addition of clathrin-mediated endocytosis inhibitors prevents EGL disruption and endothelial hyperpermeability triggered by DENV NS1. **(A)** The integrity of the EGL on HPMEC was assessed by the presence of heparan sulfate [9] surface expression (green, bottom row), as well as cathepsin L (CTSL) activity (red, top row), at 6 hpt with DENV NS1 (5 μg/ml) in the presence or absence of Pitstop 2 (12.5 μM), or Pitstop 2 alone, at 37°C, as visualized via IFA. Nuclei were stained with Hoechst (blue). Images (20X; scale bars, 50 μm) are representative of 3 independent experiments. **(B)** Quantification of MFI in **(A)** from 3 independent experiments expressed as either fold change of cathepsin L activity from Pitstop 2 control values (left) or normalized percentage of heparan sulfate from Pitstop 2 control values [10]. Error bars indicate SEM. **, p<0.01; ***, p<0.001. **(C)** HPMEC were grown on Transwell semi-permeable membranes (0.4 μm pore size), and the apical chamber was treated with either DENV NS1+ (5 μg/ml) and DMSO (blue squares); the dynamin inhibitor Dynasore (25 μM) alone (purple diamonds) or Dynasore and NS1 (orange triangles); or the clathrin-mediated endocytosis inhibitor Pitstop 2 (12.5 μM) alone (light green triangles) or Pitstop 2 and NS1 (pink circles). A TEER assay was used to evaluate the effect of NS1 and inhibitors on endothelial permeability at indicated time-points over 48 hours. (^) represents change of medium. Relative TEER values from 2 independent experiments performed in duplicate are plotted. Error bars indicate standard error of the mean (SEM).

To test whether DENV NS1 internalization is required for EGL disruption, we evaluated the effect of Pitstop 2 treatment on cathepsin L activation and heparan sulfate degradation (Fig 5A and 5B). Pretreatment of HPMEC with Pitstop 2 prevented the activation of cathepsin L and subsequent EGL degradation by WT DENV NS1, as demonstrated by decreased cathepsin L activity and reduced heparan sulfate on the surface of HPMEC, respectively (Fig 5A and 5B). We next asked whether DENV NS1 internalization is required to trigger endothelial hyperpermeability *in vitro*. Dynasore, a common inhibitor of endocytosis, antagonizes the small GTPase dynamin, which is required for clathrin-mediated endocytosis [32, 33]. Pretreatment of HPMEC with either Dynasore or Pitstop 2 abolished DENV NS1-triggered endothelial hyperpermeability as measured by TEER (Fig 5C and S10 Fig). To confirm that these inhibitors blocked clathrin-mediated endocytosis in our system, we measured the capacity of both Pitstop 2 and Dynasore to block internalization of transferrin, a protein well appreciated to be taken up via clathrin-mediated endocytosis [34]. As expected, Pitstop2 and Dynasore, but not the DMSO vehicle control, blocked internalization of transferrin into HPMEC (S11A and S11B Fig).

**Fig 6:**
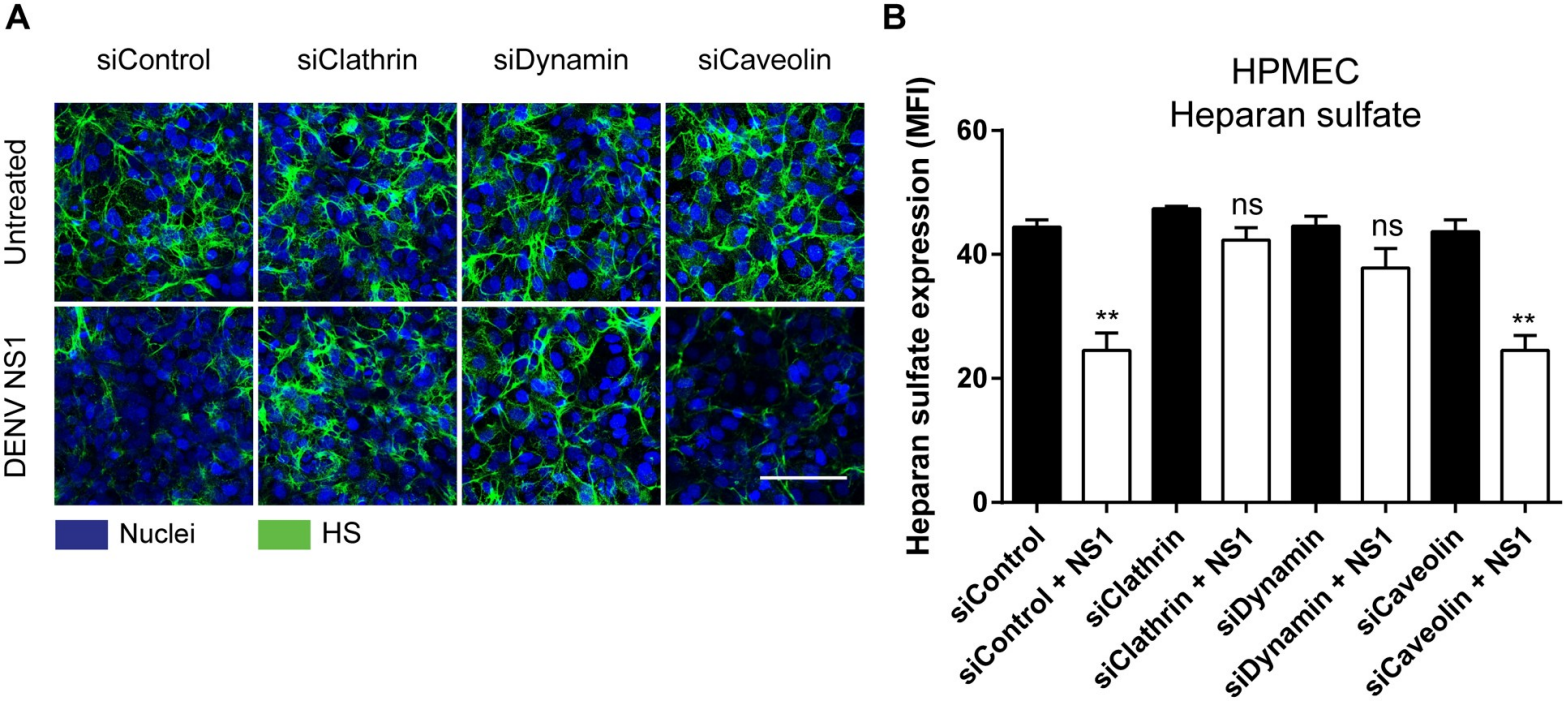
Clathrin and dynamin, but not caveolin are required for the NS1-mediated disruption of the EGL. **(A)** HPMEC were transfected with the indicated siRNA for 72 hours then treated with 5 ug/ml DENV2 NS1. The integrity of the EGL on HPMEC was assessed 6 hours post NS1-treatment by the presence of heparan sulfate (green) [9] on the cell surface. Nuclei were stained with Hoechst (blue). Images (20X; scale bars, 50 μm). **(B)** Quantification of MFI in (A) from 3 independent experiments. ns = not significant, **, P < 0.01.

Because chemical inhibitors can have off-target effects, we genetically knocked down clathrin, dynamin, and caveolin to confirm the role of clathrin-mediated endocytosis in DENV NS1 EGL disruption. Utilization of small interfering RNAs (siRNA) targeting either the clathrin heavy chain, dynamin I and II (I/II), or caveolin-1 mRNA enabled us to knock down expression of the proteins encoded by these transcripts. Knocking down clathrin and dynamin, but not caveolin, abolished NS1-mediated EGL disruption, as determined by heparan sulfate surface expression (Fig 6A and 6B, S12 Fig). To confirm that our siRNA knock-down experiments successfully blocked clathrin-mediated and caveolin-mediated endocytosis in our system, we conducted internalization assays in these cells using transferrin as well as human serum albumin, which is known to be internalized via caveolin-mediated endocytosis [35]. As expected, transferrin internalization was significantly inhibited by knocking down clathrin and dynamin expression, while internalization of human serum albumin was only inhibited by knocking down caveolin expression in cells (S11C, S11D and S12 Figs), confirming the specificity of our siRNA system. These results indicate that NS1 is internalized via clathrin-mediated endocytosis, which is critical for NS1-induced hyperpermeability and EGL degradation.

**Fig 7:**
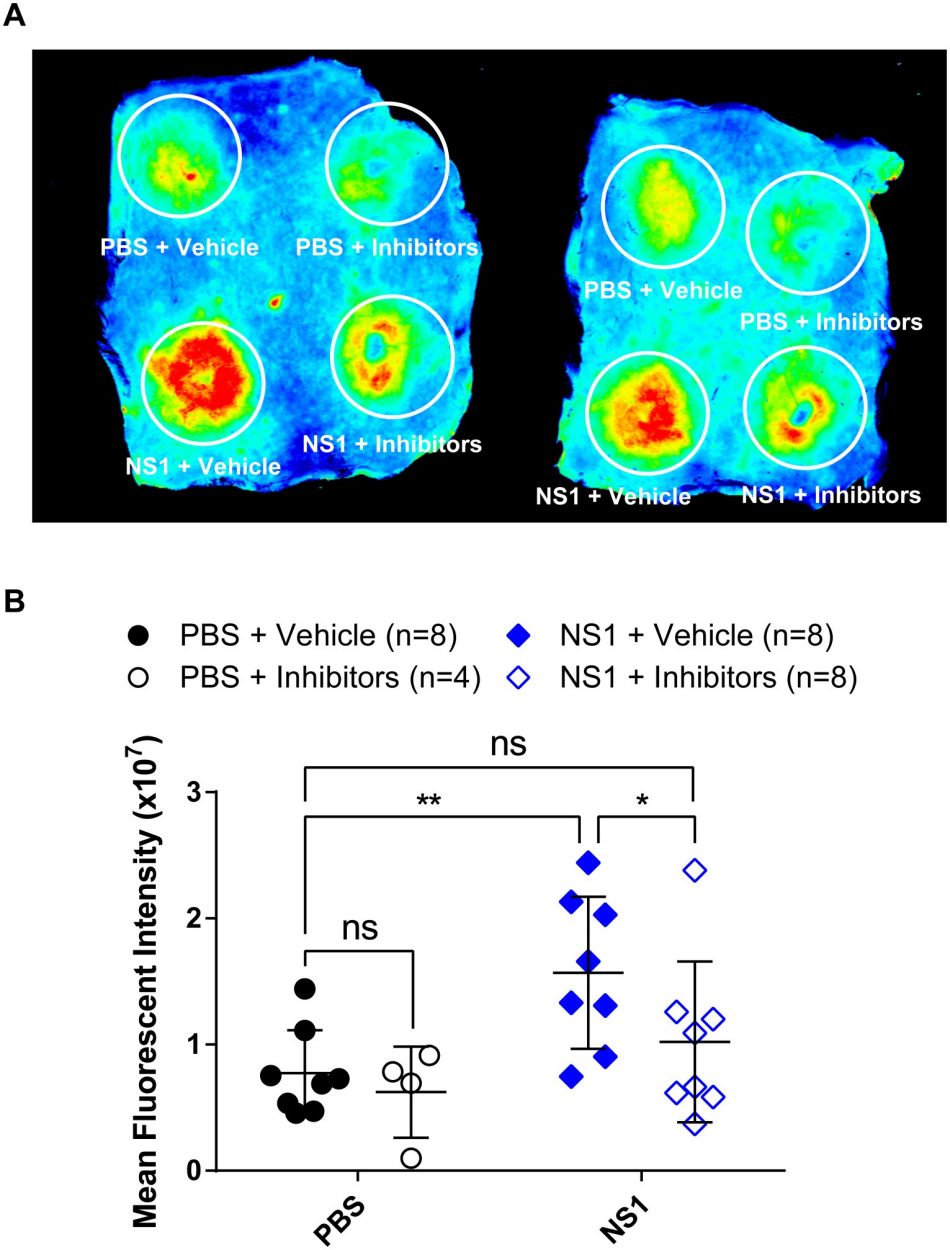
DENV2 NS1-triggered vascular leak in the dorsal dermis of mice is prevented by clathrin-mediated endocytosis inhibitors. Hair was removed from the dorsal dermis of mice, and mice were allowed to recover for 3 days. On the day of the assay, retro-orbital injections of Alexa Fluor 680-conjugated dextran were administered, followed by intradermal injections of PBS plus vehicle (DMSO, top left circle), PBS plus inhibitor cocktail (top right circle), 15 μg DENV2 NS1 plus the DMSO vehicle (bottom left circle), and 15 μg DENV2 NS1 plus inhibitors (bottom right circle). The dermis from each mouse was collected and processed two hours post-injection. **(A)** Representative image of the dorsal dermis from 2 mice following the fluorescent dextran assay. **(B)** Dermises were scanned using a fluorescent detection system (LI-COR Odyssey CLx Imaging System) at a wavelength of 700 nm, and extravasated fluorescent dextran was quantified in tissue using Image Studio software (LI-COR Biosciences). Data represent the quantification of mean fluorescent intensity from mice in (A): PBS plus DMSO vehicle (closed black circles, n = 8); PBS plus inhibitors (open black circles, n = 4); DENV2 NS1–15 μg plus DMSO vehicle (closed blue diamonds, n = 8); and DENV2 NS1–15 μg plus inhibitors (open blue diamonds, n = 8). Data in (B) represent mean +/- SD and were collected from two independent experiments. All DENV NS1 proteins used in this experiment were purchased from the Native Antigen Company. A nonparametric Mann-Whitney U test was used to determine significance between treatment groups. ns = not significant, *, p < 0.05; **, p < 0.01. The cocktail of inhibitors was composed of Pitstop 2 (0.25 mg/ml) and Dynasore (0.5 mg/ml) to antagonize local clathrin-mediated endocytosis.

To determine whether clathrin-mediated endocytosis is required for NS1 to trigger vascular leak *in vivo*, we utilized a mouse model of localized leak in the dermis [10]. In this assay, NS1 or control proteins are injected intradermally (ID) into the depilated backs of mice; up to four different conditions can be tested per mouse. Fluorescently labeled soluble dextran (Alexa Fluor 680-dextran) is then administered intravenously, and accumulation of Alexa Fluor 680-dextran in the backs of mice (where ID injections of NS1 or controls occurred) is monitored at 2 hours post-dextran injection. We injected mice with NS1 or PBS mixed with a vehicle control or with a cocktail of Pitstop 2 and Dynasore to antagonize clathrin-mediated endocytosis locally (4 conditions total). Injection with NS1 mixed with a vehicle control showed significantly greater fluorescent signal (i.e., leakage) compared to the PBS conditions or NS1 mixed with the clathrin-mediated endocytosis inhibitor cocktail, suggesting that this cocktail blocked NS1-mediated leak in the dermis. These data suggest that NS1-triggered leakage *in vivo*, in the mouse dermis, requires clathrin-mediated endocytosis (Fig 7A and 7B).

### The N207 glycosylation site of NS1 is conserved and is required to trigger endothelial hyperpermeability by multiple flaviviruses

**Fig 8:**
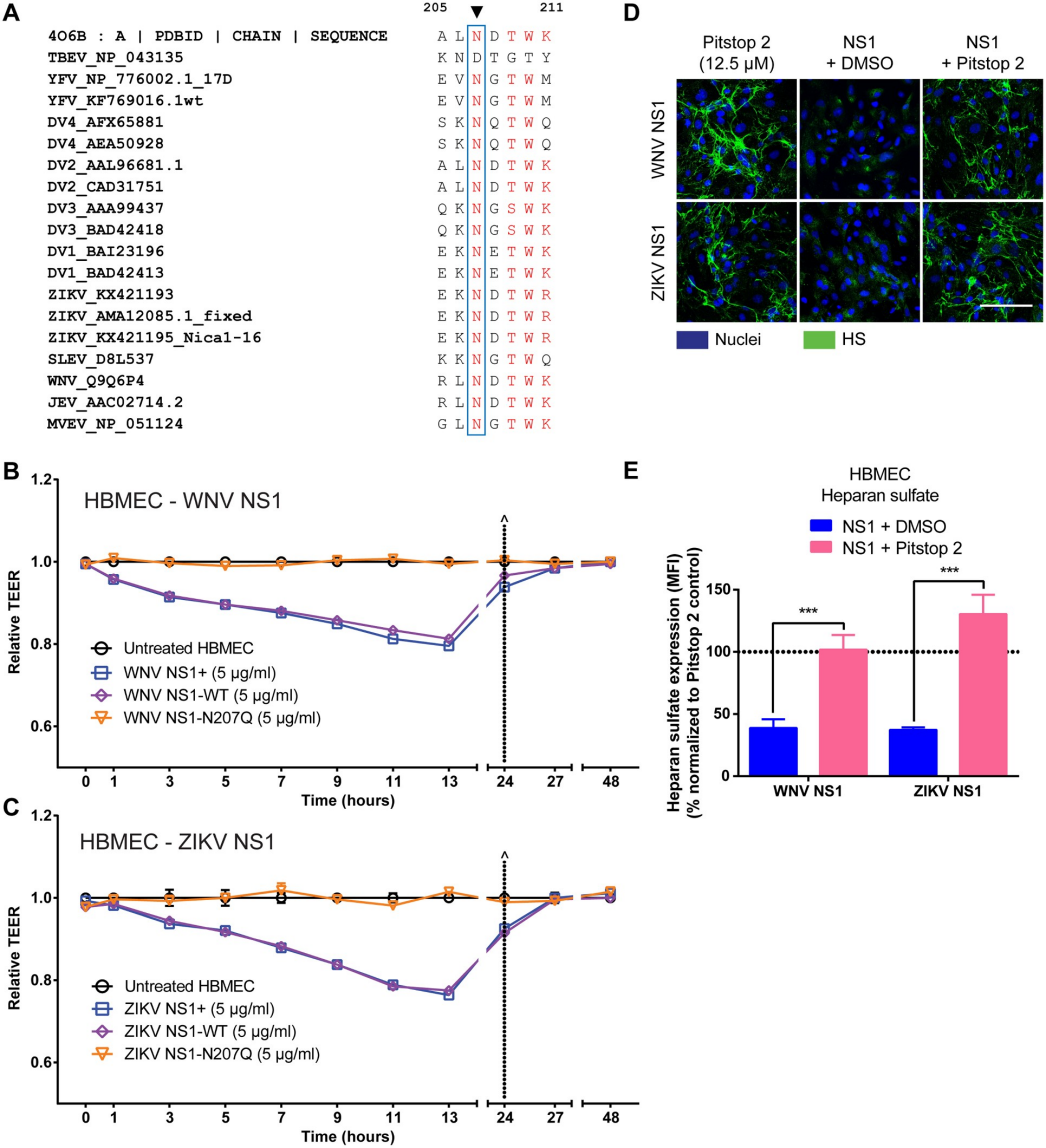
Mutation of the N207 glycosylation site abrogates endothelial hyperpermeability induced by NS1 proteins from WNV and ZIKV. (**A**) Amino acid sequence alignment of flavivirus NS1 sequences indicate that N207 glycosylation site is highly conserved. Accession numbers for each NS1 protein sequences are listed after the corresponding flavivirus names. Sequence analysis was performed using MultAlin and recreated in Excel. The amino acid sequence of NS1 from aa 205–211 is shown. **(B-C)** Human brain microvascular endothelial cells (HBMEC) were grown on Transwell semi-permeable membranes (0.4 μm pore size), and WNV NS1 **(B)** or ZIKV NS1 **(C)** proteins (5 μg/ml) were added to the apical chamber (NS1+, commercially purchased NS1, blue squares; NS1-WT, produced in-house, purple diamonds; NS1-N207Q mutant, orange triangles). A TEER assay was used to evaluate the effect of these NS1 proteins on endothelial permeability at indicated time-points over 48 hours. (^) represents change of medium. Relative TEER values from 2 independent experiments performed in duplicate are plotted. Error bars indicate SEM. **(D)** The integrity of the EGL on HBMEC was assessed by the presence of heparan sulfate surface expression (green) at 6 hpt with WNV NS1 or ZIKV NS1 (5 μg/ml) in the presence or absence of Pitstop 2 (12.5 μM) at 37°C, as visualized via IFA. Nuclei were stained with Hoechst (blue). Images (20X; scale bars, 50 μm) are representative of 3 independent experiments. (**E**) Quantification of MFI in (D) from 3 independent experiments. ***, p < 0.001.

Given the importance of the N207 glycosylation site for DENV NS1-mediated endothelial hyperpermeability, we investigated its prevalence among related viruses and found a high level of conservation of this site in multiple flaviviruses (Fig 8A). We then expanded our study to examine the role of the N207 glycosylation site of NS1 on the endothelial permeability of HBMEC in two closely related neurotropic flaviviruses–WNV and ZIKV. We first generated the WNV and ZIKV NS1-WT and NS1-N207Q mutant recombinant proteins **(**S1 Table). All proteins were secreted equivalently and appeared stable throughout the purification/dialysis procedure; further, all proteins were determined to be pure by silver staining and in a hexameric/oligomeric structure by native gels (S13A–S13F Fig). We found that WT WNV and ZIKV NS1 (commercial or produced in-house) triggered endothelial hyperpermeability as measured by TEER, indicating that WT NS1 proteins are capable of inducing endothelial hyperpermeability in HBMEC [36, 37] (Fig 8B and 8C and S14A and 14B Fig). In contrast to the WT NS1 proteins, both the WNV NS1-N207Q and ZIKV NS1-N207Q mutants failed to trigger endothelial hyperpermeability of HBMEC, suggesting that the N207 glycosylation site is a critical determinant for endothelial hyperpermeability induced by NS1 from multiple flaviviruses (Fig 8B and 8C and S14A and S14B Fig). To determine whether the N207 residue is also required for internalization of NS1 from related flavivirus such as WNV and ZIKV, as it is for DENV NS1, we conducted a binding/internalization assay of NS1-WT and NS1-N207Q from DENV, WNV, and ZIKV using HPMEC and HBMEC. In this internalization assay, we measured colocalization of NS1 with Rab5 and interpreted Rab5-positive NS1 puncta to represent binding and internalization while Rab5-negative puncta represent binding only. As observed previously [37], DENV NS1-WT bound well to both HPMEC and HBMEC while WNV and ZIKV NS1 only efficiently bound to HBMEC, and this was also the case for the NS1-N207Q mutants (S15A–S15C Fig). Further, within HBMEC, to which ZIKV and WNV NS1 efficiently bound, the NS1-WT proteins but not the NS1-N207Q mutants of DENV, WNV, and ZIKV were efficiently internalized (S15A–S15C Fig). These data suggest that like DENV NS1, the N207-glycosylation site is critical for internalization of WNV and ZIKV NS1 proteins. We next investigated whether clathrin-mediated endocytosis was a common pathway for flavivirus NS1-mediated pathogenesis. We found that Pitstop 2 blocked heparan sulfate shedding on HBMEC induced by WT WNV NS1 and WT ZIKV NS1 (Fig 8D–8E). Taken together, these results indicate that internalization of NS1 via a clathrin-mediated endocytosis pathway is a conserved mechanism for NS1-induced endothelial hyperpermeability for multiple flaviviruses.

## Discussion

**Fig 9:**
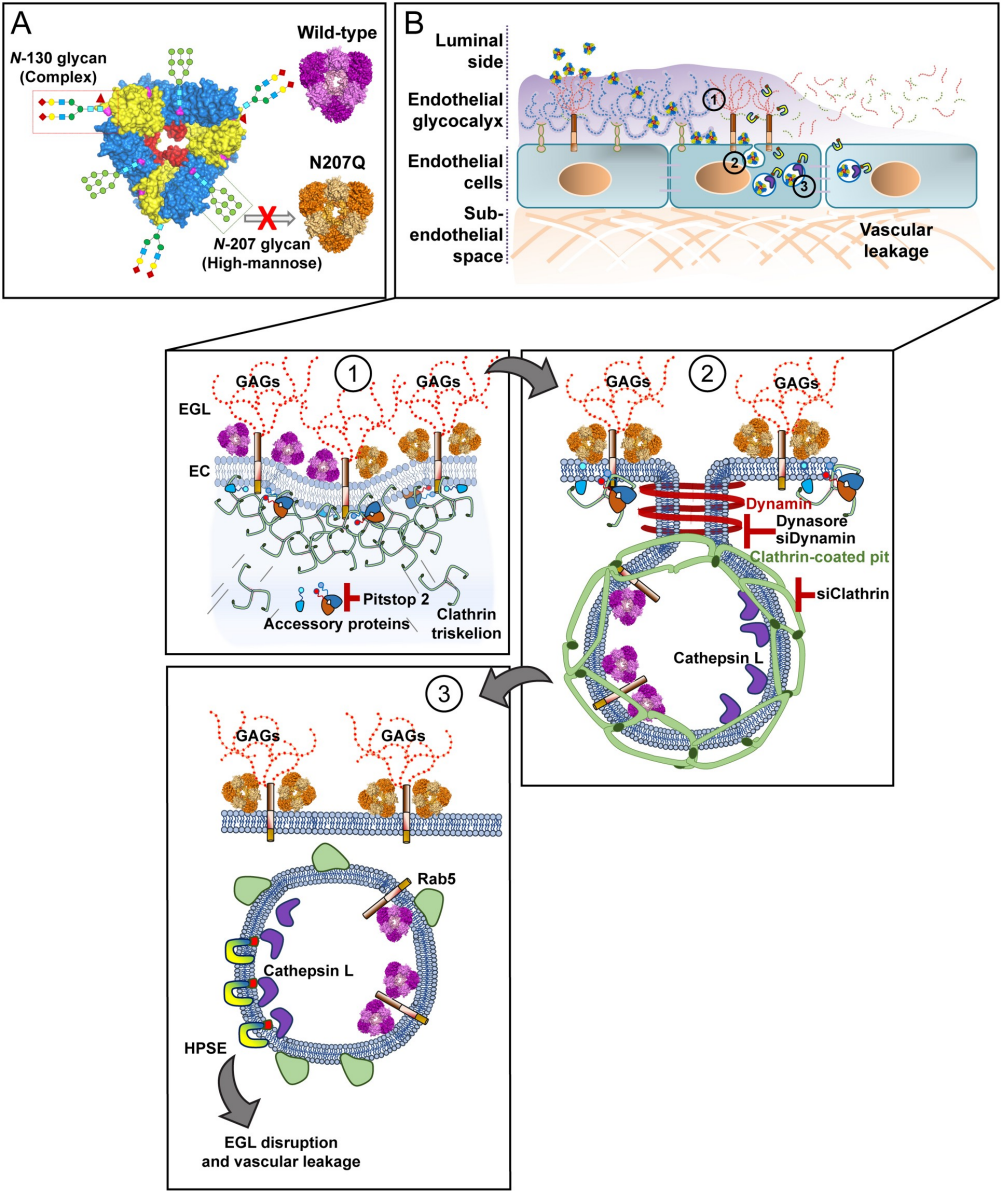
A single N-glycosylation mutant (N207Q) abrogates clathrin mediated-endocytosis of different flavivirus NS1 proteins in human endothelial cells, which is required to trigger EGL disruption and vascular leakage. **(A)** The flavivirus NS1 proteins are secreted as hexamers and contain two glycosylation sites (DENV, ZIKV, JEV, and YFV; aa 130 and 207) or three glycosylation sites (WNV; aa 130, 175, and 207). **(B)** Overview of NS1 effects on endothelial cells. **(B1)** Both NS1-WT and the NS1-N207Q mutant bind to glycosaminoglycans (GAGs; e.g., heparan sulfate) on endothelial cells (ECs), decorating the endothelial glycocalyx layer (EGL) and the cell surface. **(B2)** Both WT and the N207Q mutant of NS1 trigger recruitment of clathrin triskelion proteins to the plasma membrane. However, only the WT protein can induce maturation and scission of the clathrin-coated pit in a dynamin-dependent manner, while the N207Q mutant appears to remain sequestered on the cell surface. The use of specific chemical inhibitors for clathrin (i.e., Pitstop2) and dynamin (i.e. Dynasore) and siRNA transfection with siClathrin and siDynamin prevent NS1 internalization into HPMEC. **(B3)** This internalization process results in NS1 colocalization with an early endosome marker, Rab5. Clathrin-mediated endocytosis of NS1 (WT) triggers remodeling of the EGL after activation of endosomal-resident proteases such as cathepsin L, which induces activation of the endoglycosidase heparanase, inducing degradation of heparan sulfate on the EGL, leading to endothelial hyperpermeability and vascular leakage.

In this study, we demonstrate that the N207 glycosylation site of DENV NS1 is essential for inducing EGL degradation and hyperpermeability of human endothelial cells. We found that the DENV NS1-N207Q mutant binds to cells at similar levels as WT DENV NS1 but is retained on the cell surface, in contrast to WT DENV NS1, which is rapidly internalized. Additionally, internalization of WT DENV NS1 was dynamin- and clathrin-dependent, but independent of caveolin. Further, clathrin-mediated endocytosis of WT DENV, WNV, and ZIKV NS1 was required for endothelial hyperpermeability and EGL disruption. Taken together, these results indicate that the N207 glycosylation site is required for endothelial cell internalization via clathrin-mediated endocytosis and endosomal trafficking of NS1, which is necessary for the activation of enzymes such as cathepsin L that lead to EGL degradation and increased endothelial permeability (Fig 9A and 9B).

Using a transgenic expression system, it was previously shown that the N130 glycan of DENV NS1 was required for stabilization of the secreted NS1 hexamer whereas the N207 glycan facilitated secretion and extracellular protein stability [19]. In our system, the N-glycan at position 130 was required for protein secretion while the N-glycan at position 207 was dispensable for protein secretion and stability, in contrast to Somnuke et al. These differences could be explained by the methods used to produce and purify NS1 proteins between the two studies (*e*.*g*., a different signal sequence mediating secretion and a different strategy and methodology utilized for protein purification). In our current mammalian protein expression system utilizing 293F suspension cells, the DENV NS1-N207Q mutant was efficiently secreted; however, neither the DENV NS1-N130Q mutant nor the double mutant N130Q+N207Q was efficiently secreted, suggesting that the N130 N-glycan site is essential for NS1 secretion. These observations are supported by a previous study that demonstrated that ablation of the homologous glycosylation sites in yellow fever virus NS1 resulted in viral mutants with impaired NS1 secretion [12]. Due to this technical limitation, the N130Q and N130Q+N207Q constructs were not further characterized in this study. However, based on the significant homology at NS1-N130 across all flaviviruses, it is possible that NS1-N130 may also carry out important pathological functions, which urge further investigation.

We have previously shown that NS1 activates key endothelial cell-intrinsic pathways, including the sialidase and cathepsin L/heparanase pathways, and that these contribute to NS1-induced hyperpermeability of human endothelial cells [9]; however, how these enzymes become activated remain unknown [9]. Our results here provide evidence that internalization of NS1 via clathrin-mediated endocytosis is required for the activation of these pathogenic processes and demonstrate that NS1 localization in endosomes and lysosomes, where cathepsin L is localized, correlates with cathepsin L activation. Further studies defining the biochemical interactions required for this activation are needed and are an area of active investigation.

Our data suggest that NS1 binding and internalization are distinct steps in triggering endothelial hyperpermeability. Because both WT DENV NS1 and DENV NS1-N207Q are able to bind to HPMEC, but only WT DENV NS1 is internalized, we hypothesize that NS1 initially binds to heparan sulfate moieties on endothelial cells [25], independently of the N207 N-glycan. DENV NS1 may then interact with an additional binding partner(s) in a manner dependent on the N207 N-glycan, which leads to internalization, endosomal trafficking, and activation of cathepsin L and/or sialidases. Why is the N207 N-glycosylation site critical for internalization of DENV NS1? First, the N207 N-glycan may be required for engaging a specific receptor on endothelial cells, either directly through a physical interaction or indirectly through a conformational change. Based on the available crystal structure of NS1, amino acid 207 is not predicted to be surface-exposed [14]. This observation favors a model where a conformational change of NS1 allows this N-glycan to be exposed and mediate an interaction required for internalization or where the N-glycan itself mediates a conformational change of NS1 that allows another domain to mediate an interaction required for internalization. Second, glycosylation patterns have been shown to be important for lateral diffusion of proteins on the plasma membrane [38, 39]. Thus, an alternate possibility is that the N207 N-glycan is required for NS1 to migrate to the proper microdomain to interact with functional components on the lipid membrane required for internalization. Furthermore, NS1 is secreted from DENV-infected cells predominantly as a soluble hexameric barrel-shaped, high-density lipoprotein with a hydrophobic core containing lipid cargos such as triglycerides, cholesteryl esters, and phospholipids [15, 40, 41]. The functional importance, if any, of these lipid cargos is yet to be determined. A third possibility for the role of the N207 N-glycan in NS1 internalization is that this sugar moiety may be important for acquisition of specific lipid cargos that play a role during NS1 binding to endothelial cells or even internalization; thus, further investigation of the lipid contents of the mutant NS1 is needed to uncover a potential role of the N207 N-glycan in lipid cargo acquisition as well as a possible role of these lipid cargos in NS1-mediated pathogenesis [42].

Intriguingly, the NS1-N207Q mutant binds to cells and even recruits clathrin to the cell surface, but fails to be internalized and traffic into early endosomes. Because of these observations, an important remaining question is at what stage of the internalization process is the NS1-N207Q mutant blocked? Cell binding of NS1-N207Q alone may be sufficient to trigger the proper signaling pathway(s) to recruit clathrin to the cell surface but may not be sufficient to trigger the next stage of internalization, perhaps mediated through the engagement of the N207 N-glycan to a receptor. Previous reports of stalled clathrin-coated pits suggest that recruitment of additional factors, such as the clathrin-interacting protein epsin, are required for full maturation and budding of the endosome into the cell [30, 43]. Thus, another potential explanation may be that the N207 N-glycan is required to recruit additional factors to the clathrin-coated pits that promote internalization. An understanding of the role that other cellular factors, like epsins, play in the maturation of NS1-containing clathrin-coated pits and subsequent internalization is needed to fully elucidate the internalization defect of NS1-N207Q.

Clathrin-mediated endocytosis of NS1 may have implications beyond NS1-triggered endothelial hyperpermeability. It is known that DENV invades cells via endocytosis [44], and pharmacologically blocking clathrin-mediated endocytosis retards DENV replication [45]. Further, a recent study demonstrated that DENV NS1 interacts with DENV structural proteins, which modulates infectious viral particle production [46]. In light of our finding that NS1 is internalized via clathrin-mediated endocytosis, NS1 could potentially play a role in mediating clathrin-mediated endocytosis of flavivirus virions. Further studies are needed to elucidate, and potentially differentiate, clathrin-mediated endocytosis of virions via the E protein and/or NS1.

Regarding overall pathogenic mechanisms, our investigation specifically focuses on a cell-intrinsic pathway by which NS1 mediates the disruption of the EGL, but flavivirus-induced vascular dysfunction is a complex and multifactorial process. It can be mediated through the NS1 endothelial cell-intrinsic pathway [9, 10, 36, 37], through production of proinflammatory cytokines by immune cells induced by either the virus or NS1 [11, 47], through NS1-activated platelets [48], or even potentially through direct viral infection leading to apoptosis of endothelial cells [49, 50]. Though endothelial cells can be infected *in vitro* by DENV, findings from autopsy studies suggest that this does not occur *in vivo* [22, 51, 52]. ZIKV infects human endothelial cells *in vitro* and fetal endothelial cells in a mouse model [4, 53, 54], suggesting another pathway by which ZIKV may trigger endothelial dysfunction. Dissecting the relative contributions of each of these pathways in flavivirus-induced pathology *in vivo* is critical for designing appropriate therapeutics that could be used to treat flavivirus-induced vascular dysfunction.

All flaviviruses must disseminate from the blood into specific tissues, where they replicate to high levels. They can do this by infecting migrating immune cells, direct infection of endothelial cells [55], or through dysfunctional endothelial cell barriers [36, 56]. Our data indicate that the cell-intrinsic endothelial dysfunction pathway is triggered not only by DENV NS1, but also by neurotropic WNV and ZIKV NS1 in HBMEC. Our previous study demonstrates that NS1 from multiple flaviviruses (including DENV, WNV, and ZIKV) mediates endothelial dysfunction and vascular leak in a tissue-specific manner reflecting viral tropism [37]. We speculate that this conserved NS1-mediated cell-intrinsic endothelial dysfunction pathway mediates virus dissemination of different flaviviruses into an appropriate tissue, promoting viral replication.

In summary, our current study not only identifies the mechanism of internalization of secreted NS1 by human endothelial cells via a dynamin- and clathrin-mediated, caveolin-independent endocytosis pathway, but also pinpoints a single amino acid, the N207 glycosylation site of NS1, as a key determinant of NS1-mediated endothelial hyperpermeability. Mutation of N207 prevents NS1 internalization by human endothelial cells and inhibits disruption of the EGL and endothelial barrier function *in vitro* and *in vivo*. Further, our work identifies N207 of NS1 as a conserved functional residue among multiple flaviviruses (ZIKV and WNV, in addition to DENV) that is essential for flavivirus NS1-mediated endothelial dysfunction via clathrin-mediated endocytosis in human cells. This new molecular insight into the mechanism of flavivirus NS1-triggered endothelial hyperpermeability is crucial for understanding pan-flaviviral pathogenesis, as well as for developing antiviral therapies and NS1-based vaccine approaches.

## Materials and methods

### Cloning of recombinant NS1 constructs

A panel of recombinant DENV2 (strain 16681), WNV (NY99), and ZIKV (Nica1-16) NS1 proteins was generated, including wild-type (WT) and site-specific mutant constructs, and the primers utilized are listed in S1 Table. Amplicons consisting of the WT DENV or WNV NS1 gene preceded by the CD33 signal sequence and followed by a 6xHis-tag were a gift from M.S. Diamond (Washington University in St. Louis), along with the pmab vector. The WT ZIKV NS1 gene fragment was designed according to the ZIKV Nica1-16 strain sequence [4] and synthesized by Bio Basic Inc. To introduce mutations, the Phusion High-Fidelity DNA Polymerase (New England BioLabs; NEB) was used to perform overlap-extension PCR according to the manufacturer’s instructions. XbaI and MluI-HF restriction endonucleases (NEB) were used to digest the PCR amplicons and the pmab vector. A Quick Ligation (NEB) was performed to ligate the inserts with the vector, and 5-alpha Competent *E*. *coli* cells (NEB) were used for transformation. Colonies were selected based on expected XbaI and MluI-HF double digestion profiles and correct Sanger sequencing results.

### Cell culture

FreeStyle 293F suspension cells (Thermo Fisher Scientific) derived from human embryonic kidney cells were cultured in FreeStyle 293 Expression medium with 1% penicillin/streptomycin at 37°C and 8% CO_2_ and were maintained at 0.15–1.2 x 10^6^ cells/ml on a cell shaker at 135 rpm. The HPMEC-ST1.6r line was kindly donated by Dr. J.C. Kirkpatrick at Johannes Gutenberg University, Germany, and was grown using the EGM-2 bullet kit (Clonetics, Lonza) and maintained as previously described [8]. HBMEC were donated by Dr. Ana Rodriguez at New York University and maintained using endothelial cell medium with growth supplement (ScienCell Research Labs).

### 293F transfection and NS1 protein purification

Transfections were performed using FreeStyle 293F suspension cells and FreeStyle MAX transfection reagent (Thermo Fisher Scientific) according to the manufacturer’s protocol, and supernatant containing NS1 were collected 48 hours post-transfection. To prevent protein degradation, a tablet of EDTA-free protease inhibitor cocktail (Roche Life Sciences) was added to every 150 ml of supernatant collected. Cell supernatants were stored at -80°C prior to purification. NS1-containing supernatants were then thawed and concentrated in an Amicon filter with a 100-KDa size cutoff (UFC9100, Millipore). The 6xHis-tagged recombinant DENV NS1 proteins were purified by batch method using nickel nitrilotriacetic acid (Ni-NTA) resin agarose beads (Thermo Fisher Scientific). In brief, Ni-NTA resin agarose beads were first equilibrated in binding buffer (20 mM sodium phosphate, 500 mM sodium chloride, 20 mM imidazole, pH 7.4), and then supernatants containing NS1 were diluted 1:2 in 2X binding buffer and added to the previously equilibrated Ni-NTA resin agarose beads. NS1-containing supernatants were allowed to bind to the Ni-NTA resin for 30 minutes at 4°C rocking end-over-end. Once binding was completed, the resin was washed 4X in wash buffer (20 mM sodium phosphate, 500 mM sodium chloride, 30 mM imidazole, pH 7.4). Concentrated NS1 was eluted from the Ni-NTA resin using an imidazole-containing elution buffer (20 mM sodium phosphate, 500 mM sodium chloride, and 200 mM imidazole, pH 7.4). This purified NS1 stock was then subjected to dialysis against 1X PBS for 48 hours at 4°C. Typical concentrations of NS1 stocks obtained ranged from 0.1–0.5 mg/ml. The Pierce BCA Protein Assay Kit (Thermo Fisher Scientific) was used to quantify the purified recombinant proteins.

### Western blot

For the Endo H/PNGase F digestion Western blots, protein stability assays Western blots, NS1 internalization kinetic Western blots, siRNA knockdown Western blots, and ZIKV/WNV native-PAGE Western blots total cell lysates or recombinant proteins were collected in protein sample buffer (0.1 M Tris [pH 6.8], 4% SDS, 4 mM EDTA, 286 mM 2-mercaptoethanol, 3.2 M glycerol, 0.05% bromophenol blue) and then resolved by SDS-PAGE. The same protocol and buffer were used for native-PAGE but without SDS. The resolved proteins were then transferred onto nitrocellulose membranes and probed with primary antibodies diluted in TBS/0.1% Tween 20 (TBST) containing 5% skim milk, rocking overnight at 4°C. Secondary antibodies were diluted in TBST with 5% skim milk for 1-hour rocking at room temperature. After each antibody incubation, membranes were washed three times for 5 minutes each with TBST. Membranes were visualized using ECL reagents on a ChemiDoc system with Image Lab software from Bio-Rad. All antibodies were probed by the above method except rabbit anti-Caveolin1 and rabbit anti-DynaminI/II which were probed in TBST with 5% bovine serum albumin according to the manufacturer’s instructions. The following antibodies were used: mouse anti-6xHis (MA1-21315, Thermo Scientific), anti-NS1 monoclonal antibody (7E11), rabbit anti-clathrin heavy chain (ab172958, Abcam), rabbit anti-Dynamin I/II (2342S, Cell Signaling), rabbit anti-caveolin 1 (3267, Cell Signaling), mouse anti-βactin (sc-47778, Santa Cruz Biotechnology), mouse anti- αtubulin (ab4074, Abcam), goat anti-mouse HRP (405306, Biolegend), and donkey anti-rabbit HRP (406401, Biolegend). For the N-glycan digestion Western blots, 1 μg of denatured NS1s were digested with 750 units Endo H (NEB) or 500 units PNGase F (NEB) treatment at 37°C for 1 hour according to manufacturer’s protocol. For the NS1 stability assays 100 ng of protein were mixed with EGM-2 media (Lonza) and incubated in a tissue-culture incubator for the times indicated in the figures.

For all other Western blots, SDS-PAGE gels were transferred semi-dry onto a polyvinylidene difluoride (PVDF) membrane for 7 minutes [57] at 25V. The membrane was blocked and incubated with primary anti-6xHis-tag antibody overnight. After washing, a goat anti-mouse IgG secondary antibody conjugated to Alexa Fluor 680 (Thermo Fisher Scientific) was added at a dilution of 1:5,000 for 1 hour, and blots were imaged using the LI-COR Odyssey imaging system. Anti-NS1 mAb 7E11 (generously donated by R. Putnak at the Walter Reed Army Institute of Research) was used for detection of DENV NS1.

### Size-exclusion chromatography

0.25 milligrams (500 ul of a 0.5 mg/ml stock) of purified and dialyzed NS1-WT and NS1-N207Q were injected into a Superose 6 Increase 10/300 GL column (GE Life Sciences) previously equilibrated with 1X PBS. Validation of peaks, detected by UV light, were done by evaluating 2.5 ul of a given fraction by SDS-PAGE and probing with a NS1-speficic monoclonal antibody (7E11).

### Enzyme-linked Immunosorbent Assay (ELISA)

NS1 capture ELISA: MaxiSorp ELISA plates (Thermo Scientific Nunc) were coated with 50 μl of an anti-NS1 specific monoclonal antibody (7E11) (5 μg/ml) and incubated overnight at room temperature. The next day the plate was blocked with 1% BSA in PBS for 1 hour then washed twice with 1X PBS. Then 50 ul of NS1 containing supernatant was added to wells and incubated for 1 hour at room temperature. Plates were then washed 3 times in PBS + 0.1% Tween20 and incubated for 1 hour at room temperature with another NS1-specific monoclonal antibody (2B7). After an additional three washes in PBS + 0.1% Tween20 a peroxidase-labeled goat anti-mouse secondary antibody (Jackson ImmunoResearch) at 0.5 μg/ml in 1% BSA-PBS was added to the wells and incubated for an additional hour. After 3 additional washing steps, the assay was developed using an ABTS-HRP substrate kit (KPL), according to the manufacturer’s specifications.

NS1 direct ELISA: To examine the conformational status of the recombinant NS1 proteins used in this study, we performed a NS1 direct ELISA using a panel of recombinant NS1 proteins (NS1+, NS1-WT, and NS1-N207Q) and anti-NS1 monoclonal antibodies (mAb) that interacts with the soluble NS1 protein in a conformational-dependent manner. Briefly, ELISA plates (Nunc, Thermo Scientific) were coated overnight at 4°C with a single concentration of NS1 (200 ng/mL) diluted in sterile DPBS (1X, Gibco) under two conditions: native (no SDS) or denaturing (SDS 0.1%, boiling at 99°C for 5 minutes). The next day, plates were washed twice using wash buffer (PBS + 0.05% Tween-20) and blocked using blocking buffer (3% bovine albumin, BSA in washing buffer) for 45 minutes at room temperature (RT). Then, plates were probed individually with anti-NS1 mAbs (0.25 μg/mL) which bind NS1 proteins under denaturing conditions (e.g. 7E11 and 2B7) or native conditions (e.g. 9NS1). All recombinant NS1 proteins in this study contain a 6xHis-tag. The anti-6xHis-tag mAb (0.25 μg/mL, Thermo Scientific) was used as positive control for NS1 detection. A peroxidase conjugated goat-anti mouse IgG (H+L) antibody (Jackson Immunoresearch Inc.,)(1:5000) and TMB (3,3′,5,5′-Tetramethylbenzidine, Sigma) were used to develop the assay. Plates were allowed to develop for 30 minutes at room temperature and were then stopped with a solution of sulfuric acid (H_2_SO_4_, 2N). Plates were read at a 450 nm wavelength using a microplate reader.

### Transendothelial electrical resistance (TEER)

To measure the functional effects of the recombinant NS1 proteins, HPMEC and HBMEC were cultured in duplicate wells (80,000 cells in 0.3 ml) in the apical chamber of 24-well Transwell polycarbonate membrane inserts (Transwell permeable support, 0.4 μm, 6.5 mm insert; Corning Inc.) and incubated with 5 μg/ml NS1 protein (WT or mutants, 1.5 μg total protein). TEER was measured as previously described [8, 9]. Resistance values were measured in Ohms (Ω) at sequential 2-hour time-points following the addition of test proteins using an Epithelial Volt Ohm Meter (EVOM) with “chopstick” electrodes (World Precision Instruments). Untreated endothelial cells grown on Transwell inserts were used as negative untreated controls, and inserts with medium alone were used for blank resistance measurements. Relative TEER represents a ratio of resistance values (Ω) as follows: (Ω experimental condition-Ω medium alone)/(Ω non-treated endothelial cells-Ω medium alone).

### NS1 protein binding assay

Confluent HPMEC monolayers grown on gelatin-coated coverslips (0.2%, Sigma) were treated with 5 μg/ml of DENV2 NS1+ from the Native Antigen Company (Oxfordshire, UK) as a positive control, WT DENV NS1 produced in-house, or the DENV NS1-N207Q mutant and incubated for 1 hour at 37°C/4°C or for 6 hours at 37°C, depending on the experiment. To remove heparan sulfate from the cell surface, HPMEC were treated for 24 hours, prior to NS1 treatment with 0.5 units of recombinant heparanase (H3917-50 UN, Sigma). An anti-6xHis-tag antibody conjugated to DyLight 647 (Thermo Fisher Scientific) was used to detect NS1 protein bound to the cell surface. Untreated cells were used as a negative control. Images were acquired using a Zeiss LSM 710 AxioObserver-34-channel spectral detector confocal microscope and processed using ImageJ software. For MFI quantification, the threshold for each individual channel (RGB) was adjusted and converted to grayscale. Then, mean grayscale values and integrated density from selected areas were obtained, along with adjacent background readings, and plotted as mean fluorescence intensity (MFI).

### Immunofluorescent microscopy

HPMEC were grown and imaged as described above. To assess the effect of flavivirus NS1 on the integrity of EGL components such as sialic acid and heparan sulfate, HPMEC monolayers were treated with DENV WT (Native Antigen or produced in-house) or NS1-N207Q mutant proteins (5 μg/ml) and fixed with 4% paraformaldehyde (PFA) at different time-points (1, 6 hpt). A primary antibody against heparan sulfate (clone F58-10E4, Amsbio) was incubated overnight at 4°C, and detection was performed using a secondary goat anti-mouse IgM antibody conjugated to Alexa 488 (Thermo Fisher Scientific). The cell surface expression of sialic acid was visualized using the lectin wheat germ agglutinin (WGA) conjugated to Alexa 647 (Thermo Fisher Scientific) [9]. The proteolytic activity of cathepsin L was evaluated using the Magic Red assay cathepsin L detection kit (ImmunoChemistry Technologies) [9]. Nuclei were stained with Hoechst (blue). Untreated cells were used as a control for basal sialic acid and heparan sulfate expression and cathepsin L activity. Images were acquired using the Zen 2010 software (Zeiss) and analyzed with ImageJ software. For representative pictures, an area of ~1.8 μm^2^ (1.25x1.40 μm) containing ~28–30 cells was used. MFI quantification was performed as described above.

### NS1 protein internalization in HPMEC and HBMEC

To evaluate the internalization of DENV NS1 proteins in HPMEC, we used Western blot and IFA. Briefly, confluent monolayers (pre-chilled for 10 minutes at 4°C) grown on culture plates and/or glass coverslips were exposed to 10 μg/ml of different NS1 proteins (as indicated above) and incubated for 45 minutes at 4°C to facilitate NS1 protein adsorption but not internalization. Cultures were then transferred to 37°C to allow protein internalization. For the internalization kinetic experiments, cells were washed twice with PBS, before transfer to 37°C, to remove any unbound NS1. One hour later (or at the indicated times for kinetic analysis), cell supernatants were removed, and cell monolayers were rinsed 3X with PBS and detached with trypsin-EDTA, which also removed any surface-bound, non-internalized NS1 proteins from the outer cell membrane. Cells were pelleted and lysed using SDS gel loading buffer, then the cell lysate was separated using an SDS-PAGE gradient gel (4–20%) and visualized by Western blot with primary anti-NS1 mAbs (1G5.3 and 1H7.4, obtained from Paul Young, University of Queensland, Australia) [58] and secondary anti-mouse antibody conjugated to Alexa 750 (ab175740, Abcam). α-Tubulin was used as a protein loading control (anti-α-tubulin ab4074, Abcam), and Rab5 was used as an early endosome marker (ab18211, Abcam). Anti-rabbit IgG conjugated to Alexa 680 was used as secondary antibody (ab175773, Abcam). Images were acquired and processed using a LI-COR Odyssey system and ImageJ software, respectively. For the internalization Western blot, at the indicated time post temperature shift, cell monolayers were rinsed 3X with PBS and then 2X with pre-chilled acid wash buffer (glycine 100 mM, and 150mM NaCl, pH 2.5), which removed any surface-bound, non-internalized NS1 proteins from the outer cell membrane. Cells were then collected in western blot sample buffer.

Additionally, internalized NS1 (10 μg/ml) was visualized by IFA by co-staining NS1 and Rab5. Briefly, after 45 minutes at 4°C (to normalize protein adsorption), cultures were transferred to 37°C for 1 hour (or the time indicated in the figure for the kinetic analysis) to facilitate protein internalization. For the internalization kinetic experiments, cells were washed twice with PBS before transfer to 37°C, to remove any unbound NS1. Before fixation of samples within the kinetic analysis, cell supernatants were removed, and cell monolayers were rinsed 3X with PBS and then 2X with pre-chilled acid wash buffer (glycine 100 mM, and 150mM NaCl, pH 2.5), removing surface-bound NS1. After fixation with 4% PFA, samples were processed by indirect IFA and confocal microscopy imaging. For experiments using inhibitors, compounds were added to wells 30 minutes before the addition of DENV NS1 protein. Initially, a co-localization plugin analysis in ImageJ software was used to define the co-localizing spots between NS1 and Rab5 in each experimental condition. The amount of spatial overlap between the two signals (NS1 “red” and Rab5 “green”) was obtained using four different frames from the maximum projections of two RGB images based on the object-based approach (JACoP) and defined by the Manders’ Coefficient, as previously described [59]. As an additional analysis to quantify the amount of internalized NS1 protein in HBMEC and HPMEC monolayers by IFA, the number of NS1-positive (NS1^+^) puncta per cell (n = 200 cells) and those co-stained with the early endosome marker Rab5 (NS1^+^Rab5^+^) were manually counted using the multi-point tool in ImageJ.

### Z-stack video overlay of NS1 internalization

An animated single 2D projection of NS1 internalization in HPMEC was created by collapsing multiple image-sections (maximum projections) obtained from confocal Z-stack acquisition analyses. Briefly, HPMEC monolayers grown on glass coverslips were treated with DENV NS1 (WT), and the glycosylation mutant (DENV NS1-N207Q) for 1 hour at 37°C. Cell monolayers were processed for immunofluorescence assay as described above co-staining for NS1 protein and the early endosome marker Rab5. Z-stacks were acquired on a Zeiss LSM 710 inverted confocal microscope (CRL Molecular Imaging Center, UC Berkeley) using a Plan-Apochromat 40X dry objective. Projections of multiple Z-stack sections (20 μm range: 20 slices; 1 μm interval) were performed and animated using Z stack reconstruction plugin in Image J, then saved as Audio Video Interleave (AVI) format. Scale bar, 1 μm.

### NS1 colocalization with clathrin and caveolin

IFA was used to determine whether clathrin-mediated endocytosis or caveolin-mediated uptake contribute to internalization of NS1 by endothelial cells. HPMEC or HBMEC were grown and imaged as described above. Briefly, after 45 minutes at 4°C (to normalize protein adsorption), plates were transferred to 37°C for 30 minutes to facilitate protein internalization. After fixation with 4% PFA, samples were processed by IFA and confocal microscopy imaging. A colocalization plugin analysis in ImageJ software was used to define the colocalizing spots between NS1 and clathrin or caveolin in each experimental condition. The amount of spatial overlap between the two signals (NS1, red, and Clathrin, Caveolin, Rab5, green) was obtained using four different frames from the maximum projections of two RGB images based on the object-based approach (JACoP) and defined by the Manders’ Coefficient as previously described [59].

### Antibodies and inhibitors for endocytosis experiments

Primary antibodies against clathrin heavy chain (Clathrin Heavy Chain [D3C6] XP Rabbit mAb [4796S], Cell Signaling), caveolin (Caveolin-1 (D46G3) XP Rabbit mAb #3267, Cell Signaling and Anti-Caveolin-2 antibody EPR5471 [ab133484], Abcam), Rab5 (Anti-Rab5 antibody—Early Endosome Marker [ab18211], Abcam), EEA1-early endosome marker (AB2900, Abcam), cathepsin L (BMS166, eBioscience), LAMP1 (ab25630, Abcam), mouse anti-6xHis (MA1-21315, Thermo Scientific), and rabbit anti-6xHis (ab232492, Abcam), together with the secondary antibodies goat anti-mouse IgG conjugated to Alexa 647 (ab150115, Abcam), goat anti-mouse IgG conjugated to Alexa 488 (ab150117, Abcam), donkey anti-rabbit IgG conjugated to Alexa 568 (ab175470, Abcam) were used in IFA experiments. An anti-6xHis-tag mAb (HIS.H8) conjugated to DyLight 647 (Thermo Fisher Scientific) was used for NS1 protein binding assays. Additionally, cells were infected with a baculovirus Rab5-GFP overexpression system (CellLight Early Endosomes-GFP, BacMam 2.0, C10586, Thermo Fisher Scientific), according to the manufacturer’s instructions, to visualize colocalization of NS1 with Rab5. Selective inhibitors of clathrin-mediated endocytosis (Pitstop 2, Abcam) and dynamin (Dynasore, Sigma) were used in IFA experiments and/or TEER assays at concentrations that do not affect cell viability. Cell viability was determined using the Promega CellTox Green Cytotoxicity Assay following the manufacturer’s instructions. The internalization of human transferrin, an iron-binding protein well-known to be internalized via clathrin-mediated endocytosis, was used as a control to examine the effectiveness of clathrin-mediated endocytosis inhibitors. Briefly, 30 minutes post-treatment with Pitstop 2 (25 μM) and Dynasore (50 μM) at 37°C, transferrin conjugated to Alexa 568 (20 μg/ml) (T23365, Thermo Fisher Scientific) was added to HPMEC monolayers cultured on cover slips and incubated for 10 minutes at 37°C. Then, plates were immediately transferred to an ice-bed; the transferrin inoculum was removed and cell monolayers were washed twice with cold PBS, and then washed twice with pre-chilled acid wash buffer (glycine 100 mM, and 150mM NaCl, pH 2.5) to remove cell-surface associated but non-internalized transferrin [33]. After this, coverslips were processed as described above for imaging after fixation with 4% PFA.

### siRNA knock-down analysis

For transient knock-down analysis of clathrin heavy chain (HC), dynamin I/II, and caveolin-1, the siRNA-mediated knock-down system from Santa Cruz Biotechnology was used. In brief, Control siRNA-A (sc-37007), Clathrin HC siRNA (h) (sc-35067), Dynamin I/II (sc-43736), and caveolin-1 siRNA (h) (sc-29241) were resuspended in siRNA Dilution Buffer (sc-29527) according to the manufacturer’s instructions. HPMEC were then transfected with siRNAs using siRNA Transfection Reagent (sc-29528) in siRNA Transfection Medium (sc-36868) according to the manufacturer’s instructions. Seventy-two hours post-transfection, cells were assayed for knock-down efficiency by Western blot analysis and used for further experiments. Primary antibodies used for Western blot were as follows: clathrin HC (ab172958, Abcam), Dynamin I/II (2342S, Cell Signaling), caveolin-1 (D46G3) XP Rabbit mAb #3267 (Cell Signaling), and α-tubulin (ab4074, Abcam). Additionally, HPMEC monolayers transiently transfected with distinct siRNAs were analyzed for internalization of fluorescently labeled-transferrin-A568 (20 μg/ml) (T23365, Thermo Scientific) and albumin-FITC (100 μg/ml) (GTX28030, GeneTex) after incubation at 37°C for 10 minutes and 1.5 hours, respectively. The amount of internalized proteins was evaluated by IFA analyses using the same acid wash buffer method described above.

### Localized vascular leak murine model

*In vivo* NS1-induced endothelial hyperpermeability was measured using a rodent model of localized vascular leak, as previously described [10]. Briefly, the dorsal area of 6-week old female WT C57BL/6 mice (Jackson Labs) was depilated 3–4 days prior to each experiment. Hair was initially trimmed using hair clippers and then removed using Nair (Church & Dwight). Excess Nair was thoroughly wiped off using 70% ethanol and water. On the day of the experiment, DENV2 NS1 (15 μg) was incubated for 15 minutes at room temperature in the presence of 0.25 mg/ml Pitstop 2 (abcam) and 0.5 mg/ml Dynasore (abcam). NS1-inhibitor mixtures and controls were then were injected intradermally (ID) into distinct sites in the depilated dorsal skin of mice. Then, 150 μl of 10-kDa dextran conjugated with Alexa Fluor 680 (1 mg/ml; Sigma) was delivered by retro-orbital (RO) injection. Two hours after injection, mice were euthanized using isoflurane, and the dorsal skin was removed and placed in Petri dishes. Tissues were then visualized and scanned using a fluorescent detection system (LI-COR Odyssey CLx Imaging System) at a wavelength of 700 nm, and leakage surrounding the injection sites was quantified using Image Studio software (LI-COR Biosciences).

### Statistics

Statistical analyses were performed using GraphPad Prism 6 software, and all graphs were generated using Prism 6. For quantification of MFI in in Figs 2B–2E, 3B, 3D and 3F and S5B and S5C Fig, an ordinary one-way ANOVA using Dunnett’s multiple comparison test with multiple comparisons was used. For MFI quantification of IFA images in Figs 4F, 5B, 6B and 8E and S6B Fig, unpaired parametric t-tests were used to determine the significance of treatment with inhibitors or siRNA knockdown of various proteins. For dermal leak experiments in Fig 7, a nonparametric Mann-Whitney U test was used to determine significance of inhibitor treatment. For MFI quantification of IFA images in S11B and S11D Fig, ordinary one-way ANOVA analyses using Dunnett’s multiple comparison test with multiple comparisons to either the DMSO group (S11B Fig) or the untreated group (S11D Fig) were used to determine the significance of inhibitor treatment or siRNA knockdown on internalization of transferrin or albumin by HPMEC.

## Supporting information

S1 Table Related to Figs 1 and 8. Cloning and primer sequences for the DENV2, ZIKV, and WNV NS1 constructs. (DOCX)

S1 Fig Related to Fig 1. DENV WT NS1 and N-glycosylation site mutant N207Q are efficiently secreted, and the NS1-N207Q mutant lacks a high mannose N-glycan. **(A)** Supernatants from 293F cells transfected with WT DENV NS1 or N-glycosylation mutant NS1-N207Q were concentrated using Amicon Ultra filters (100-kDa), and the concentrated top fraction (Top) and flow-through (FT) were both collected. NS1 monomers were detected via Western Blot of SDS-PAGE, using an anti-6xHis-tag antibody. (**B)** Western blot of SDS-PAGE showing the expression and secretion of WT DENV NS1, NS1 glycosylation mutant (N130Q), and double mutant (N130Q+N207Q). Anti-6xHis-tag antibody was used for detection of the NS1 monomer. S/N, supernatant; CL, cell lysate; NS1+, DENV2 NS1 (Native Antigen Company); Neg, gel loading buffer only. (**C**) Western blot of purified NS1 proteins treated with Endo H or PNGase F (NEB) for 1 hour at 37°C, using an anti-6xHis-tag antibody and demonstrating absence of the high mannose N-glycan at position 207 of the NS1-N207Q mutant. -, untreated; +E, Endo H-treated; +P, PNGase F-treated; arrows indicate which N-glycan species are present for each band.
(TIF)

S2 Fig Related to Fig 1. Size-exclusion chromatography reveals a comparable size and elution profile between NS1-WT and NS1-N207Q. **(A)** Size-exclusion chromatography of 0.25 milligrams of purified and dialyzed DENV NS1-WT (black) and DENV NS1-N207Q (gray). **(B)** Western blot analysis, under denaturing conditions, revealing NS1 monomers from the indicated fractions from Panel A with NS1-WT on the top and NS1-N207Q on the bottom. Proteins are detected using an NS1-specific monoclonal antibody (7E11).
(TIF)

S3 Fig Related to Fig 1. Purified NS1-WT and NS1-N207Q exist in a comparable conformation and are equally stable over time. **(A)** NS1 direct ELISA comparing binding of three non-conformational mouse monoclonal antibodies (7E11, 2B7, and anti-6xHis) and one conformational mouse monoclonal antibody (9NS1) to NS1+, NS1-WT, or NS1-N207Q at a concentration of 200 ng/ml in native conditions (PBS) or denaturing conditions (PBS + 0.1% SDS with boiling for 5 minutes). **(B)** NS1 capture-ELISA comparing stability of 100 ng of NS1+, NS1-WT, or NS1-N207Q over time. One hundred ng of the indicated NS1 was diluted in EGM-2 tissue culture medium, mixed with 0.1% SDS or 200 ug/ml Proteinase K when indicated, and placed in a tissue culture incubator (37°C with 5% CO_2_) for the indicated times. The NS1-specific monoclonal antibody (7E11) was used to capture NS1 in the medium and another NS1-specific monoclonal antibody (2B7) was used to detect the captured NS1 proteins. **(C)** Western blot analysis of the indicated samples from panel B from an SDS-PAGE gel. NS1 was detected with a mouse anti-6xHis-tag monoclonal antibody. **(D)** Same experimental setup and Western blot analysis as Panel C but measuring the later time points indicated.
(TIF)

S4 Fig Related to Fig 1. WT NS1 but not the NS1-N207Q mutant increase the permeability of HPMEC and HBMEC monolayers. Transendothelial electrical resistance (TEER) assays were used to determine the effect of the NS1-N207Q mutant on NS1-induced hyperpermeability. TEER data here are the non-normalized raw data from Fig 1 displayed in Ohms (Ω). **(A)** HPMEC values from Fig 1E and **(B)** HBMEC values from Fig 1F.
(TIF)

S5 Fig Related to Fig 2. Mutation of the N-glycosylation site 207 prevents NS1-induced sialic acid degradation. (**A**) The binding of DENV NS1 (NS1+, Native Antigen Company), the in-house-produced DENV NS1-WT, and NS1-N207Q mutant (green) to HPMEC 1 hour post-treatment (hpt) was visualized via immunofluorescence assay (IFA). The integrity of the EGL component sialic acid (Sia) was assessed after 1 hpt at 37°C. Sia, stained with WGA-A647 (red); nuclei, stained with Hoechst (blue). Images (20X; scale bars, 50μm) are representative of two independent experiments run in duplicate. (**B**) Quantitation of A (top, NS1 binding). (**C**) Quantitation of A (bottom, sialic acid). The means ± standard error of the mean (SEM) of two individual experiments run in duplicate are shown. ns, not significant; *, p<0.05; **, p<0.01.
(TIF)

S6 Fig Related to Fig 3. NS1-WT and NS1-N207Q both require heparan sulfate to bind to the surface of HPMEC. (**A**) The binding of in-house-produced NS1-WT and the NS1-N207Q mutant (10 μg/ml) (red) to HPMEC was visualized via IFA 24 hpt with 0.5 units of recombinant heparanase; untreated cells were used as a control. The nuclei of cells are stained with Hoechst (blue). Images (20X; scale bars, 50μm) are representative of three independent experiments. (**B**) Quantitation of cell binding in A. **, p<0.01. (**C**) Heparan sulfate surface expression (green) in HPMEC 24 hpt with 0.5 units of recombinant heparanase at 37°C, as visualized via IFA. Nuclei were stained with Hoechst (blue). Images (20X; scale bars, 50 μm) are representative of 3 independent experiments.
(TIF)

S7 Fig Related to Fig 3. NS1-WT but not NS1-N207Q are internalized into HPMEC and localize to the early endosome. **(A)** Western blot analysis of HPMEC from Fig 3C treated with 10 μg/ml of the indicated NS1 and incubated at 37°C for 1.5 hours. Cells were not trypsinized to demonstrate the addition of equivalent NS1 levels to cells. NS1 was detected using an NS1-specific monoclonal antibody (7E11). **(B)** IFA analysis of HPMEC from Fig 3E not treated with NS1. NS1 signal (red) is the first panel from the left and Rab5 signal (green) is the second panel from the left. Colocalization is shown in yellow in the merged image or white in the colocalization panel (ImageJ). **(C)** IFA analysis of HPMEC cells overexpressing Rab5 (CellLight-Rab5-GFP) (green) treated with NS1 (10 μg/ml) (red). Colocalization is shown in yellow in the merged image. **(D)** IFA analysis of HPMEC treated with NS1 (10 μg/ml) (red) and stained for the early-endosome marker EEA1 (green). Colocalization is shown in yellow in the merged image. For all images, nuclei are stained with Hoechst (blue). Images (40X; scale bars, 10 μm) are representative of 2 individual experiments run in duplicate. NS1+, NS1 from the Native Antigen Company.
(TIF)

S8 Fig Related to Fig 4. WT DENV NS1 colocalizes with clathrin but not with caveolin on the surface of HBMEC. Colocalization of DENV NS1 proteins with either clathrin **(A)** or caveolin **(B)** in HBMEC was performed as described in the legend for Fig 4A, except HBMEC were plated instead of HPMEC. Images are 40X; scale bars, 5 μm. Quantification of the amount of spatial overlap between the two signals, NS1 and clathrin **(C)**, or NS1 and caveolin **(D)** in S8A Fig and S8B Fig, respectively. The means ± SEM of two individual experiments run in duplicate are shown.
(TIF)

S9 Fig Related to Fig 4. NS1 rapidly and transiently accumulates inside HPMEC, where it colocalizes with the lysosomal marker Lamp1 and cathepsin L. (**A**) Internalization of NS1 (10 μg/ml) into HPMEC was measured at the indicated time-points after allowing NS1 to adsorb to the cell surface at 4°C. After NS1 adsorption, cells were washed twice in cold PBS to remove unbound NS1 and were moved to 37°C for the indicated times. At the indicated time-points, cells were fixed, permeabilized, and visualized by IFA. Before fixation, cells were washed in a glycine-acid buffer to remove NS1 that is bound to the surface but not yet internalized. NS1 is visualized by an anti-6xHis-tag antibody (green), and nuclei of cells are stained with Hoechst (blue). Images (20X; scale bars, 50 μm) are representative of two independent experiments. (**B**) MFI quantification of internalized NS1 in A. (**C**) Same as A, except cells were collected in protein sample buffer for Western blot analysis. Before cell lysis, cells were washed in a glycine-acid buffer to remove NS1 that is bound to the surface but not yet internalized. **(D)** NS1 was allowed to internalize into HPMEC for 90 minutes (unbound NS1 was not washed away), cells were then fixed and permeabilized, and colocalization of NS1 and cellular markers was visualized by IFA. NS1 was detected with an anti-6xHis-tag antibody (green, the secondary antibody was conjugated to AF488). Lamp1 (red, the secondary antibody was conjugated to AF568); CTSL, cathepsin L (red, the secondary antibody was conjugated to AF647). Images (20X; scale bars, 25 μm) are representative of two independent experiments.
(TIF)

S10 Fig Related to Fig 5. Clathrin-mediated endocytosis is required for NS1 to trigger endothelial hyperpermeability. Transendothelial electrical resistance (TEER) assays were used to determine the effect of inhibiting clathrin-mediated endocytosis on NS1-induced hyperpermeability. TEER data here are the non-normalized raw data from Fig 5C displayed in Ohms (Ω).
(TIF)

S11 Fig Related to Figs 5–7. Chemical inhibition and siRNAs successfully antagonize clathrin- and caveolin-mediated endocytosis. Internalization of **(A-D)** transferrin-Alexa 568 and **(C, D)** albumin-FITC in HPMEC monolayers grown on coverslips and **(A, B)** treated with chemical inhibitors (30 minutes at 37°C; Pitstop 2 = 25 μM; Dynasore = 50 μM) or **(C, D)** transfected with distinct siRNAs. Internalization of **(A-D)** transferrin conjugated to Alexa-568 (20 μg/ml), to control for clathrin-mediated endocytosis, and **(C, D)** albumin-FITC (100 μg/ml), to control for caveolin-mediated endocytosis, was determined after 10 minutes and 1.5 hours at 37°C, respectively. After this time, cell monolayers were washed (4X) with an acid buffer to remove bound-non-internalized proteins. The amount of internalized transferrin and albumin was determined by confocal microscopy imaging (images: 20X; scale bars, 25 μm) and expressed as mean fluorescence intensity (MFI). The means ± SEM of two (B, D right) or three (D left) individual experiments run in duplicate are shown. ns, not significant; *, p<0.05; **, p<0.01.
(TIF)

S12 Fig Related to Fig 6. Successful knockdown of siRNA targets. Western blot analysis of cell lysates from Fig 6A and 6B and S11 Fig., to test knock-down efficiency of clathrin heavy chain, dynamin I/II, and caveolin-1. Representative image shown from 3 independent Western blots. α-Tubulin is included as a loading control.
(TIF)

S13 Fig Related to Fig 8. In-house-produced WNV and ZIKV NS1-WT and NS1-N207Q are equivalently secreted, exhibit high purity and stability, and exist primarily as putative hexamers. **(A and D)** Western blot analysis, under denaturing conditions, of supernatants from 293F cells transfected with the indicated construct. **(B and E)** Silver staining analysis after SDS-PAGE of the indicated purified NS1 proteins. Gels depict NS1 monomers. **(C and F)** Western blot analysis, in native condition, of the indicated purified NS1 proteins or commercially purchased (NS1+). For all Western blots NS1 was detected using an anti-6xHis-tag antibody.
(TIF)

S14 Fig Related to Fig 8. Flavivirus NS1-WT but not the NS1-N207Q mutant increase the permeability of HBMEC monolayers. Transendothelial electrical resistance (TEER) assays were used to determine the effect of the WNV and ZIKV NS1-N207Q mutant on NS1-induced hyperpermeability. TEER data here are the non-normalized raw data from Fig 8 displayed in Ohms (Ω). **(A)** Values from HBMEC treated with WNV NS1 from Fig 8B. **(B)** Values from HBMEC treated with ZIKV NS1 from Fig 8C.
(TIF)

S15 Fig Related to Fig 8. Flavivirus NS1 residue N207 plays a conserved role in NS1 internalization. HPMEC (**A top, B**) and HBMEC (**A bottom, C**) monolayers grown on coverslips were treated with three different flavivirus NS1 proteins (DENV, ZIKV and WNV: 10 μg/ml) including NS1-WT and the NS1-N207Q mutant. NS1 binding and internalization were examined after 1.5 hours of incubation at 37°C using confocal microscopy to detect the co-staining of NS1 protein (red) and the early endosomal marker Rab5 (green). Nuclei were stained using Hoechst (blue). Images (40X; scale bars, 5 μm) are representative of 2 independent experiments. **(B, C)** The amount of total NS1 in each endothelial cell (red staining in **A**) was expressed as NS1^+^, total puncta per cell (light blue open circles), and NS1 puncta colocalized with Rab5 (yellow staining in **A**) was expressed as NS1^+^ Rab5^+^ puncta, indicating NS1 colocalization and therefore internalization (dark blue open squares). NS1^+^ puncta were counted from a total of 200 cells collected from two independent experiments and analyzed using ImageJ. Dashed lines (light grey) and solid lines (black) represent the mean values for NS1^+^ total puncta and NS1^+^Rab5^+^ puncta, respectively.
(TIF)

S1 Movie Relating to Fig 3: DENV NS1-WT but not the DENV NS1-N207Q mutant are internalized into endothelial cells where they colocalize with Rab5. An animated single 2D projection of NS1 internalization in HPMEC created using multiple confocal z-stack images. (**S1 Movie**) After binding to the cell surface, the WT NS1 protein (in *red*, indicated by white arrowheads) moves inside the cell, where it encounters Rab5 (in *green*), which results in a coalescence signal (in *yellow*), suggesting spatial colocalization. As the Z stack images move into a deeper Z axial space, this colocalizing signal disappears, and only the red signal for NS1 persists, suggesting its mobilization into a different intracellular compartment. (S2 Movie) This process does not occur with the NS1-N207Q mutant protein, where most of the NS1 (in *red*) remain at a higher Z axial space, as little to no signal appears to colocalize with Rab5, suggesting this mutant NS1 protein is retained on the cell surface.
(MP4)

S2 Movie Relating to Fig 3: DENV NS1-WT but not the DENV NS1-N207Q mutant are internalized into endothelial cells where they colocalize with Rab5. An animated single 2D projection of NS1 internalization in HPMEC created using multiple confocal z-stack images. (S1 Movie) After binding to the cell surface, the WT NS1 protein (in *red*, indicated by white arrowheads) moves inside the cell, where it encounters Rab5 (in *green*), which results in a coalescence signal (in *yellow*), suggesting spatial colocalization. As the Z stack images move into a deeper Z axial space, this colocalizing signal disappears, and only the red signal for NS1 persists, suggesting its mobilization into a different intracellular compartment. (**S2 Movie**) This process does not occur with the NS1-N207Q mutant protein, where most of the NS1 (in *red*) remain at a higher Z axial space, as little to no signal appears to colocalize with Rab5, suggesting this mutant NS1 protein is retained on the cell surface.
(MP4)
