# Supplementary material for: Endocytosis of flavivirus NS1 is required for NS1-mediated endothelial hyperpermeability and is abolished by a single N-glycosylation site mutation
Source: PLoS Pathog. 2019 Jul 29;15(7):e1007938. doi: 10.1371/journal.ppat.1007938 (PMC6687192; doi:10.1371/journal.ppat.1007938)
Supplement: S1 Table — (DOCX) [file ppat.1007938.s001.docx]

**S1 Table.** Cloning and primer sequences for the DENV2, ZIKV, and WNV NS1 constructs.

| **Construct #** | **PCR Template** | **Primer Sequences*** |
| --- | --- | --- |
| DENV WT | DENV WT  PCR amplicons | CTGCAGAACGACGCGTGCCGCCACCATGCC |
|  |  | GGATCCCTGCAGCTCGAGTCAGTGATGGTGATGG TGATG |
| DENV N130Q | PCR1: DENV WT | GCTGTTTCGGGGCCATCAATGAGAAAGGTCTGttg ATGAGACTCTGTAGA |
|  | PCR2: DENV WT | TCTACAGAGTCTCATcaaCAGACCTTTCTCATTGAT GGCCCCGAAACAGC |
| DENV N207Q | PCR1: DENV WT | CTTTCTCTATCTTCCACGTGTCttgGAGTGCACTTT CTATC |
|  | PCR2: DENV WT | GATAGAAAGTGCACTCcaaGACACGTGGAAGATA GAGAAAG |
| DENV (N130Q+N207Q) | PCR1: N130Q | Same as N207Q primer |
|  | PCR2: N130Q | Same as N207Q primer |
| WNV WT | WNV WT  PCR amplicons | CTGCAGAACGACGCGTGCCGCCACCATGCC |
|  |  | GGATCCCTGCAGCTCGAGTCAGTGATGGTGATGG TGATG |
| WNV N207Q | PCR1: WNV WT | CTT CCA CGT ATC TTG GAG CCT GCT TTC |
|  | PCR2: WNV WT | GAA AGC AGG CTC CAA GAT ACG TGG AAG |
| ZIKV WT | ZIKV WT  synthesized fragment | CTGCAGAACGACGCGTGCCGCCACCATGCC |
|  |  | GGATCCCTGCAGCTCGAGTCAGTGATGGTGATGG TGATG |
| ZIKV N207Q | PCR1: ZIKV WT | CAG CCA TGT GTC TTG CTT CTC ACT CTC |
|  | PCR2: ZIKV WT | GAG AGT GAG AAG CAA GAC ACA TGG CTG |

# S1 Table. Related to Fig 1 and Fig 8. Cloning and primer sequences for the DENV2, ZIKV, and WNV NS1 constructs.

* Underlined letters indicate the restriction enzyme sites of MluI (ACGCGT) and XhoI (CTCGAG); lower case letters highlight nucleotide changes where the point mutations were introduced.
