# Supplementary figures and images for: Endocytosis of flavivirus NS1 is required for NS1-mediated endothelial hyperpermeability and is abolished by a single N-glycosylation site mutation
Source: PLoS Pathog. 2019 Jul 29;15(7):e1007938. doi: 10.1371/journal.ppat.1007938 (PMC6687192; doi:10.1371/journal.ppat.1007938)

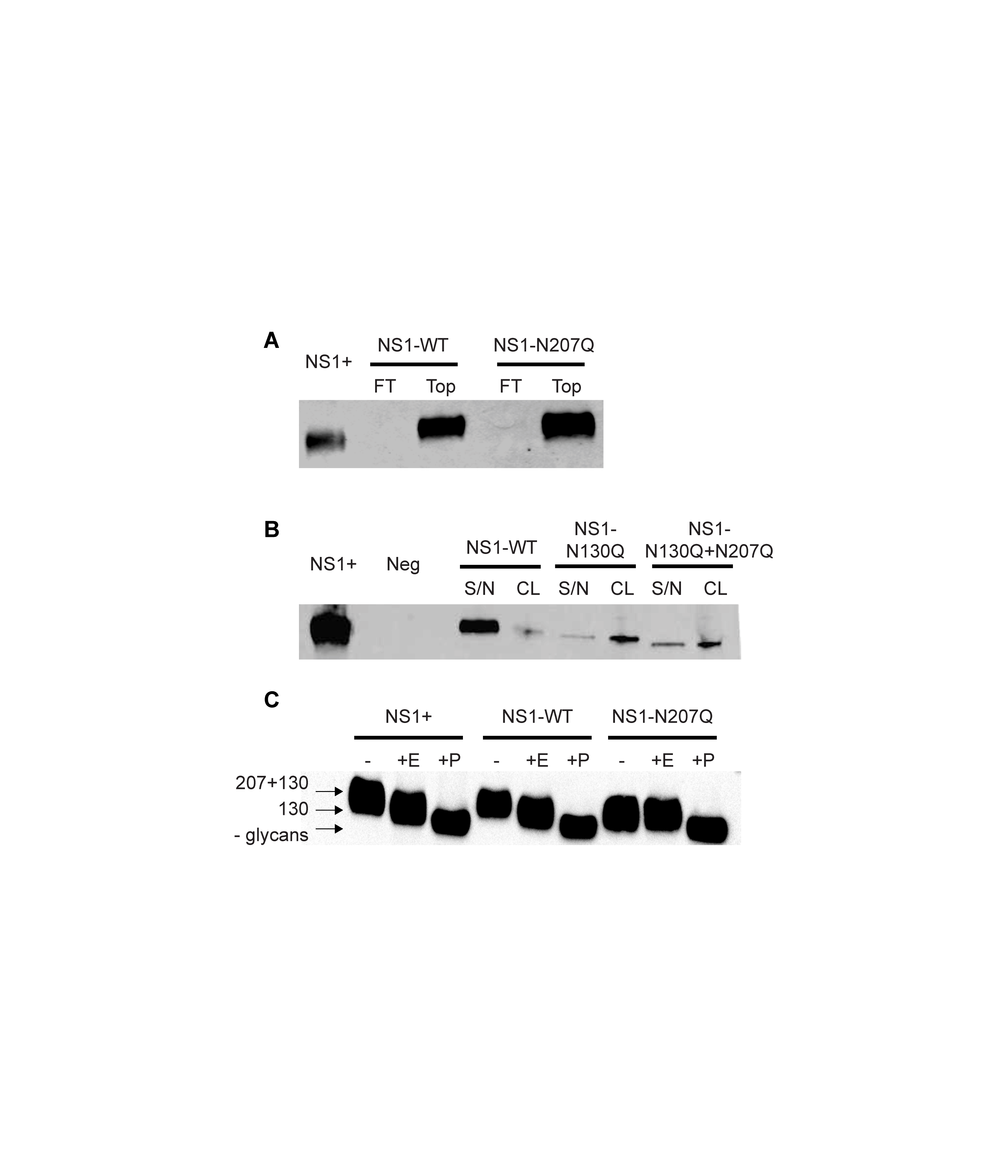

Supplement: S1 Fig — (A) Supernatants from 293F cells transfected with WT DENV NS1 or N-glycosylation mutant NS1-N207Q were concentrated using Amicon Ultra filters (100-kDa), and the concentrated top fraction (Top) and flow-through (FT) were both collected. NS1 monomers were detected via Western Blot of SDS-PAGE, using an anti-6xHis-tag antibody. (B) Western blot of SDS-PAGE showing the expression and secretion of WT DENV NS1, NS1 glycosylation mutant (N130Q), and double mutant (N130Q+N207Q). Anti-6xHis-tag antibody was used for detection of the NS1 monomer. S/N, supernatant; CL, cell lysate; NS1+, DENV2 NS1 (Native Antigen Company); Neg, gel loading buffer only. (C) Western blot of purified NS1 proteins treated with Endo H or PNGase F (NEB) for 1 hour at 37°C, using an anti-6xHis-tag antibody and demonstrating absence of the high mannose N-glycan at position 207 of the NS1-N207Q mutant. -, untreated; +E, Endo H-treated; +P, PNGase F-treated; arrows indicate which N-glycan species are present for each band. (TIF) [file ppat.1007938.s002.tif]

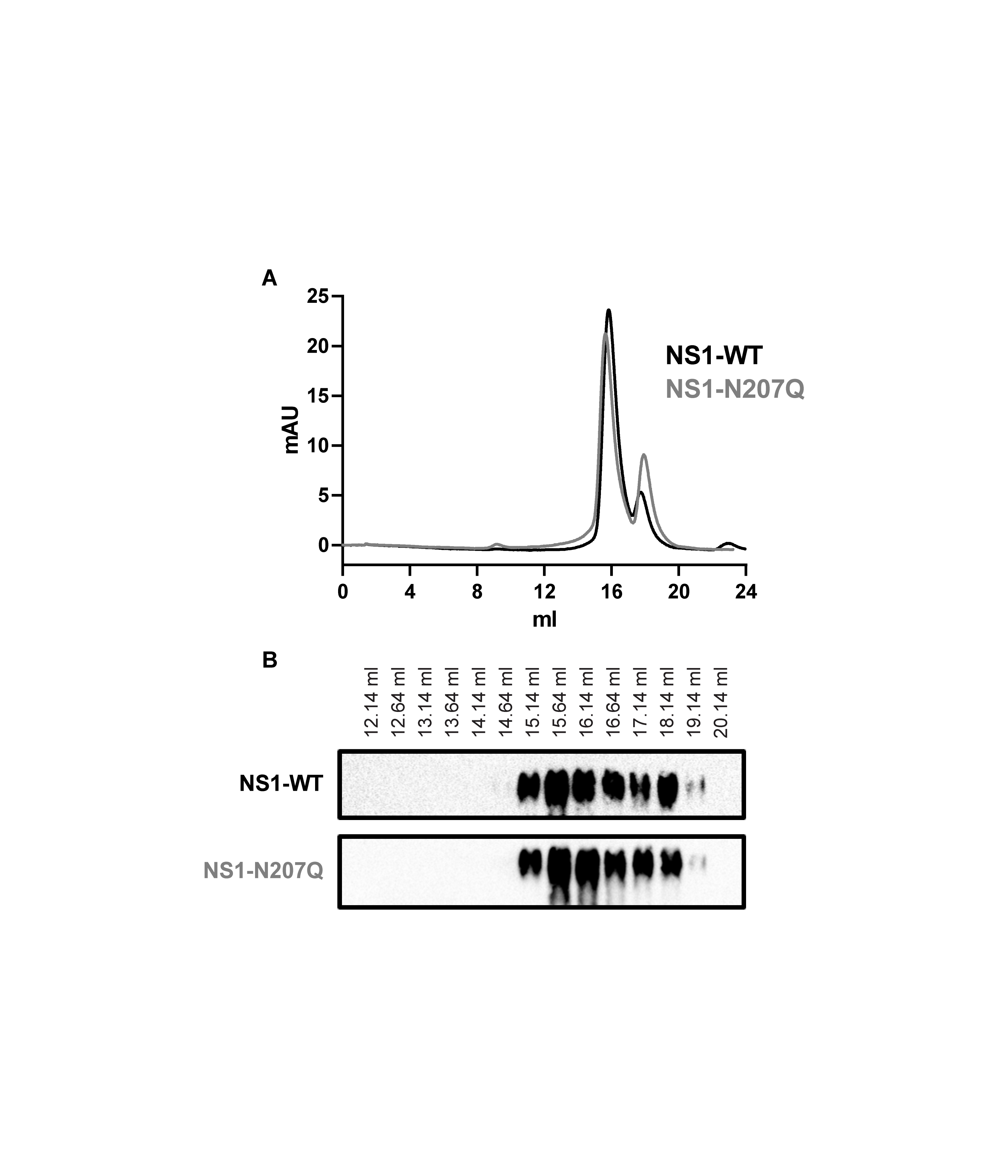

Supplement: S2 Fig — (A) Size-exclusion chromatography of 0.25 milligrams of purified and dialyzed DENV NS1-WT (black) and DENV NS1-N207Q (gray). (B) Western blot analysis, under denaturing conditions, revealing NS1 monomers from the indicated fractions from Panel A with NS1-WT on the top and NS1-N207Q on the bottom. Proteins are detected using an NS1-specific monoclonal antibody (7E11). (TIF) [file ppat.1007938.s003.tif]

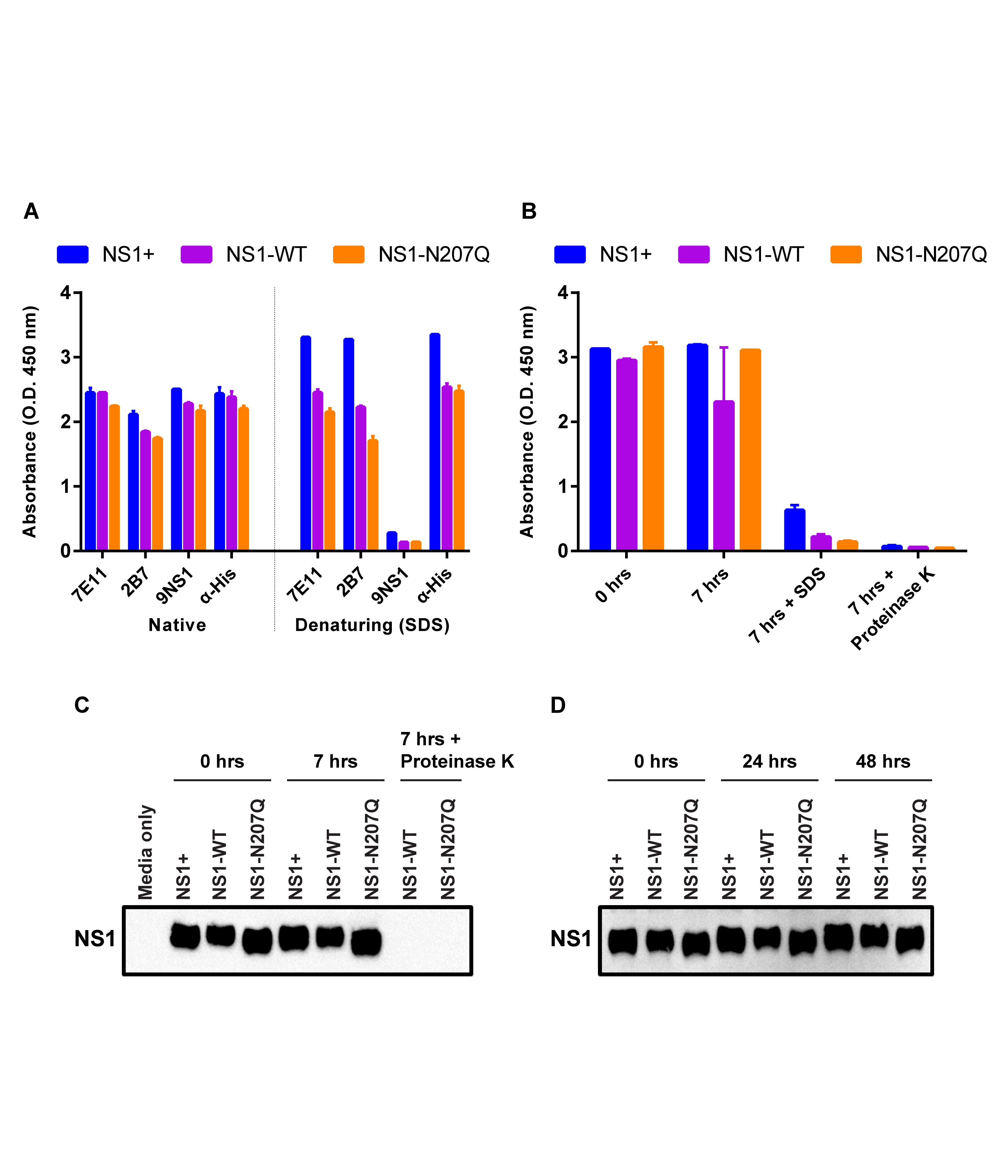

Supplement: S3 Fig — (A) NS1 direct ELISA comparing binding of three non-conformational mouse monoclonal antibodies (7E11, 2B7, and anti-6xHis) and one conformational mouse monoclonal antibody (9NS1) to NS1+, NS1-WT, or NS1-N207Q at a concentration of 200 ng/ml in native conditions (PBS) or denaturing conditions (PBS + 0.1% SDS with boiling for 5 minutes). (B) NS1 capture-ELISA comparing stability of 100 ng of NS1+, NS1-WT, or NS1-N207Q over time. One hundred ng of the indicated NS1 was diluted in EGM-2 tissue culture medium, mixed with 0.1% SDS or 200 ug/ml Proteinase K when indicated, and placed in a tissue culture incubator (37°C with 5% CO2) for the indicated times. The NS1-specific monoclonal antibody (7E11) was used to capture NS1 in the medium and another NS1-specific monoclonal antibody (2B7) was used to detect the captured NS1 proteins. (C) Western blot analysis of the indicated samples from panel B from an SDS-PAGE gel. NS1 was detected with a mouse anti-6xHis-tag monoclonal antibody. (D) Same experimental setup and Western blot analysis as Panel C but measuring the later time points indicated. (TIF) [file ppat.1007938.s004.tif]

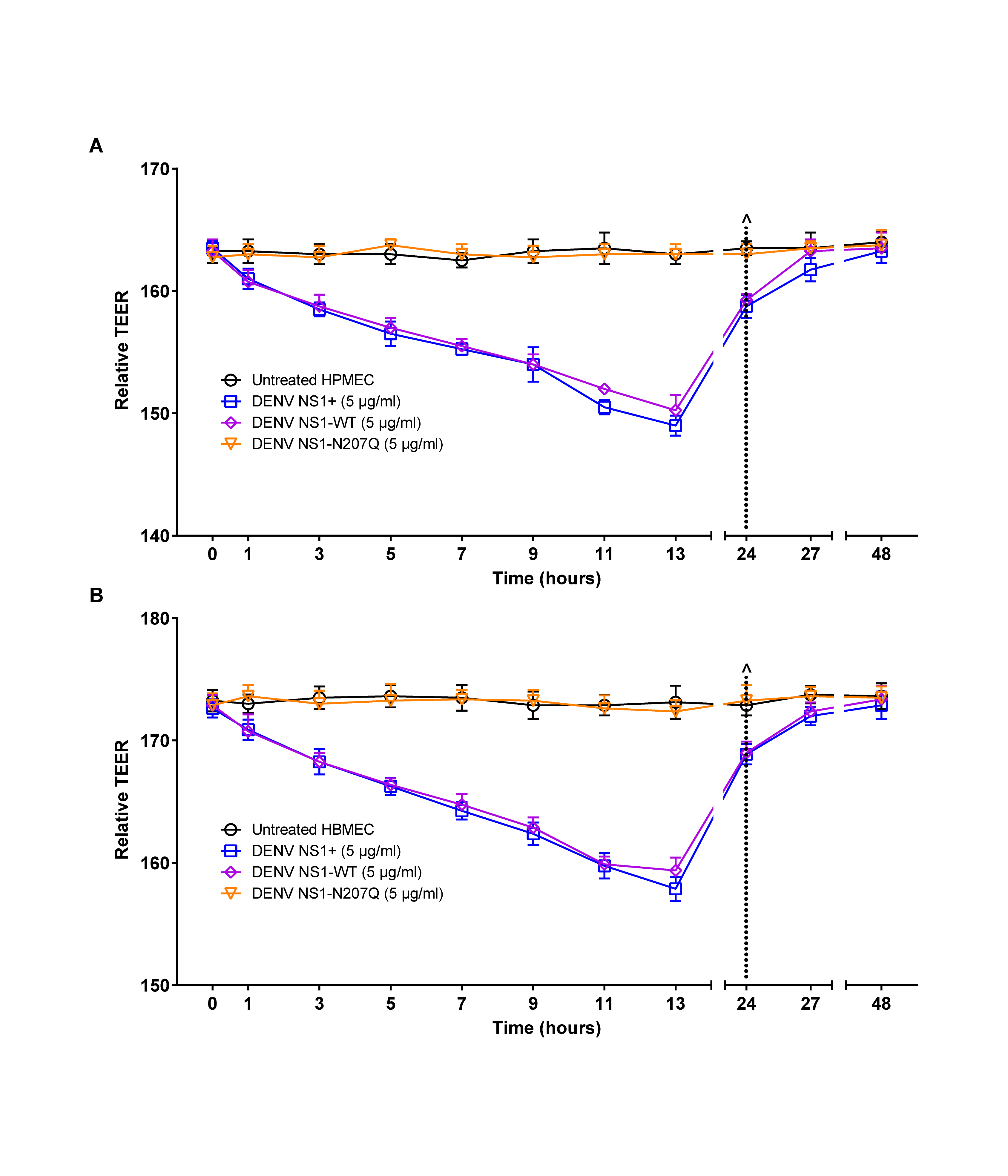

Supplement: S4 Fig — Transendothelial electrical resistance (TEER) assays were used to determine the effect of the NS1-N207Q mutant on NS1-induced hyperpermeability. TEER data here are the non-normalized raw data from Fig 1 displayed in Ohms (Ω). (A) HPMEC values from Fig 1E and (B) HBMEC values from Fig 1F. (TIF) [file ppat.1007938.s005.tif]

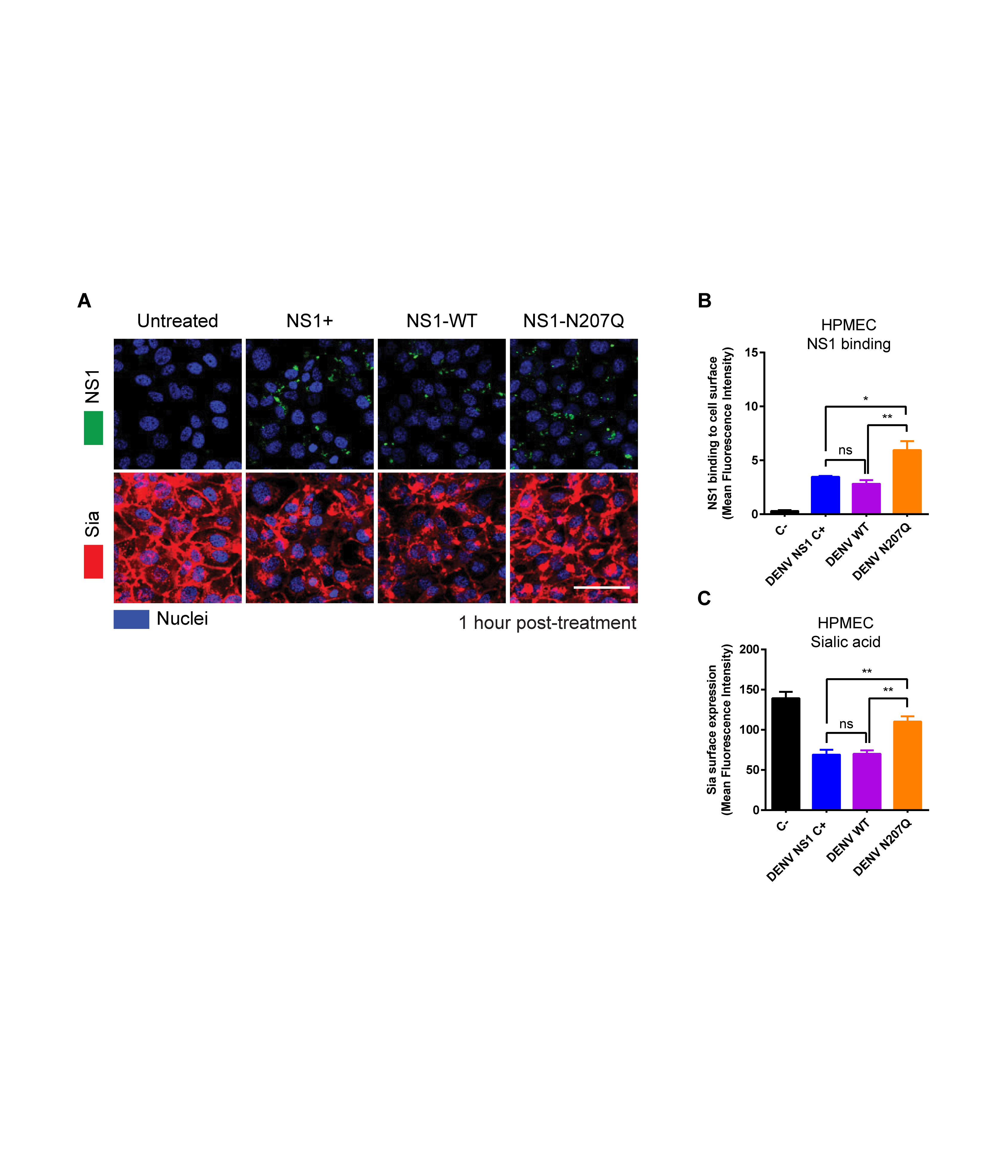

Supplement: S5 Fig — (A) The binding of DENV NS1 (NS1+, Native Antigen Company), the in-house-produced DENV NS1-WT, and NS1-N207Q mutant (green) to HPMEC 1 hour post-treatment (hpt) was visualized via immunofluorescence assay (IFA). The integrity of the EGL component sialic acid (Sia) was assessed after 1 hpt at 37°C. Sia, stained with WGA-A647 (red); nuclei, stained with Hoechst (blue). Images (20X; scale bars, 50μm) are representative of two independent experiments run in duplicate. (B) Quantitation of A (top, NS1 binding). (C) Quantitation of A (bottom, sialic acid). The means ± standard error of the mean (SEM) of two individual experiments run in duplicate are shown. ns, not significant; *, p<0.05; **, p<0.01. (TIF) [file ppat.1007938.s006.tif]

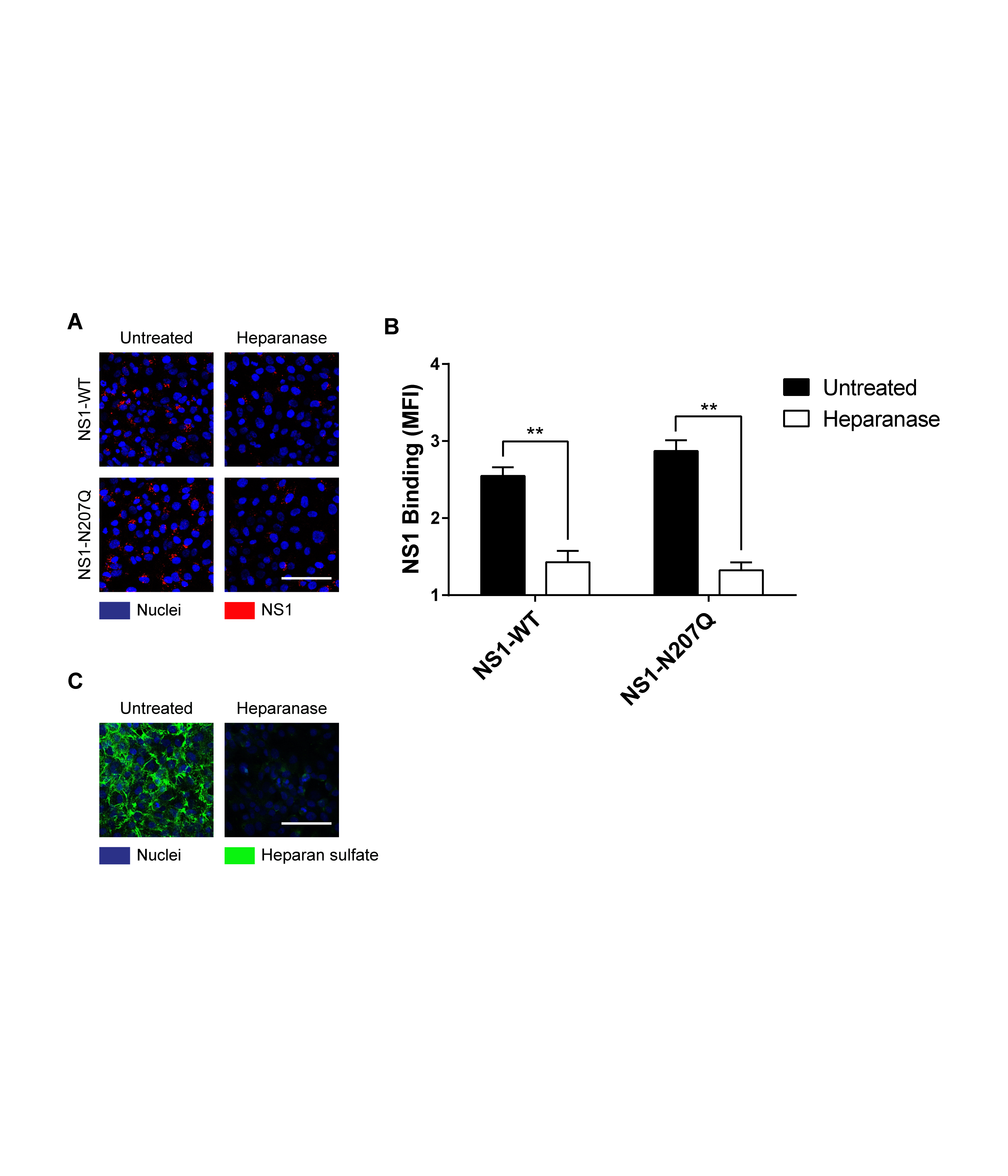

Supplement: S6 Fig — (A) The binding of in-house-produced NS1-WT and the NS1-N207Q mutant (10 μg/ml) (red) to HPMEC was visualized via IFA 24 hpt with 0.5 units of recombinant heparanase; untreated cells were used as a control. The nuclei of cells are stained with Hoechst (blue). Images (20X; scale bars, 50μm) are representative of three independent experiments. (B) Quantitation of cell binding in A. **, p<0.01. (C) Heparan sulfate surface expression (green) in HPMEC 24 hpt with 0.5 units of recombinant heparanase at 37°C, as visualized via IFA. Nuclei were stained with Hoechst (blue). Images (20X; scale bars, 50 μm) are representative of 3 independent experiments. (TIF) [file ppat.1007938.s007.tif]

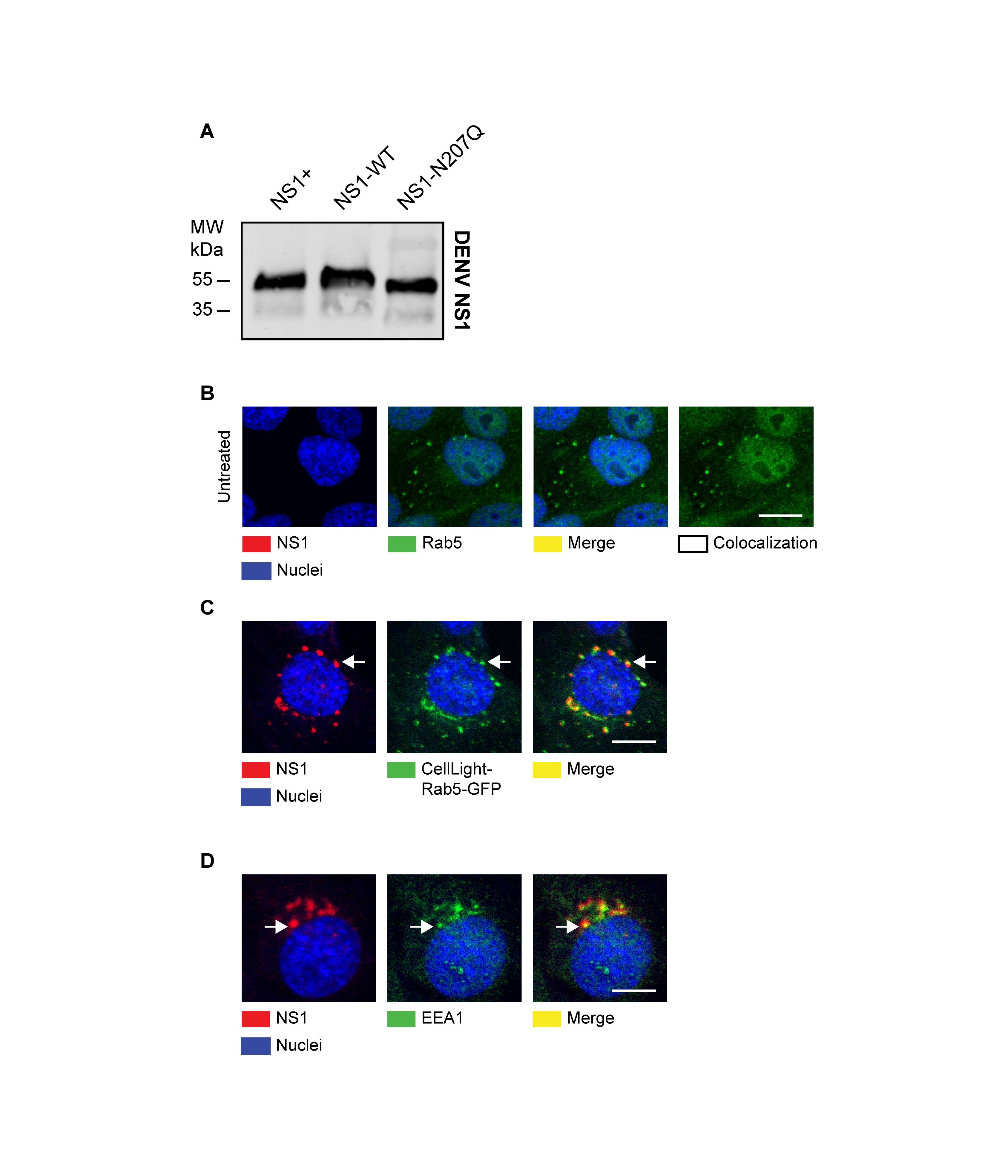

Supplement: S7 Fig — (A) Western blot analysis of HPMEC from Fig 3C treated with 10 μg/ml of the indicated NS1 and incubated at 37°C for 1.5 hours. Cells were not trypsinized to demonstrate the addition of equivalent NS1 levels to cells. NS1 was detected using an NS1-specific monoclonal antibody (7E11). (B) IFA analysis of HPMEC from Fig 3E not treated with NS1. NS1 signal (red) is the first panel from the left and Rab5 signal (green) is the second panel from the left. Colocalization is shown in yellow in the merged image or white in the colocalization panel (ImageJ). (C) IFA analysis of HPMEC cells overexpressing Rab5 (CellLight-Rab5-GFP) (green) treated with NS1 (10 μg/ml) (red). Colocalization is shown in yellow in the merged image. (D) IFA analysis of HPMEC treated with NS1 (10 μg/ml) (red) and stained for the early-endosome marker EEA1 (green). Colocalization is shown in yellow in the merged image. For all images, nuclei are stained with Hoechst (blue). Images (40X; scale bars, 10 μm) are representative of 2 individual experiments run in duplicate. NS1+, NS1 from the Native Antigen Company. (TIF) [file ppat.1007938.s008.tif]

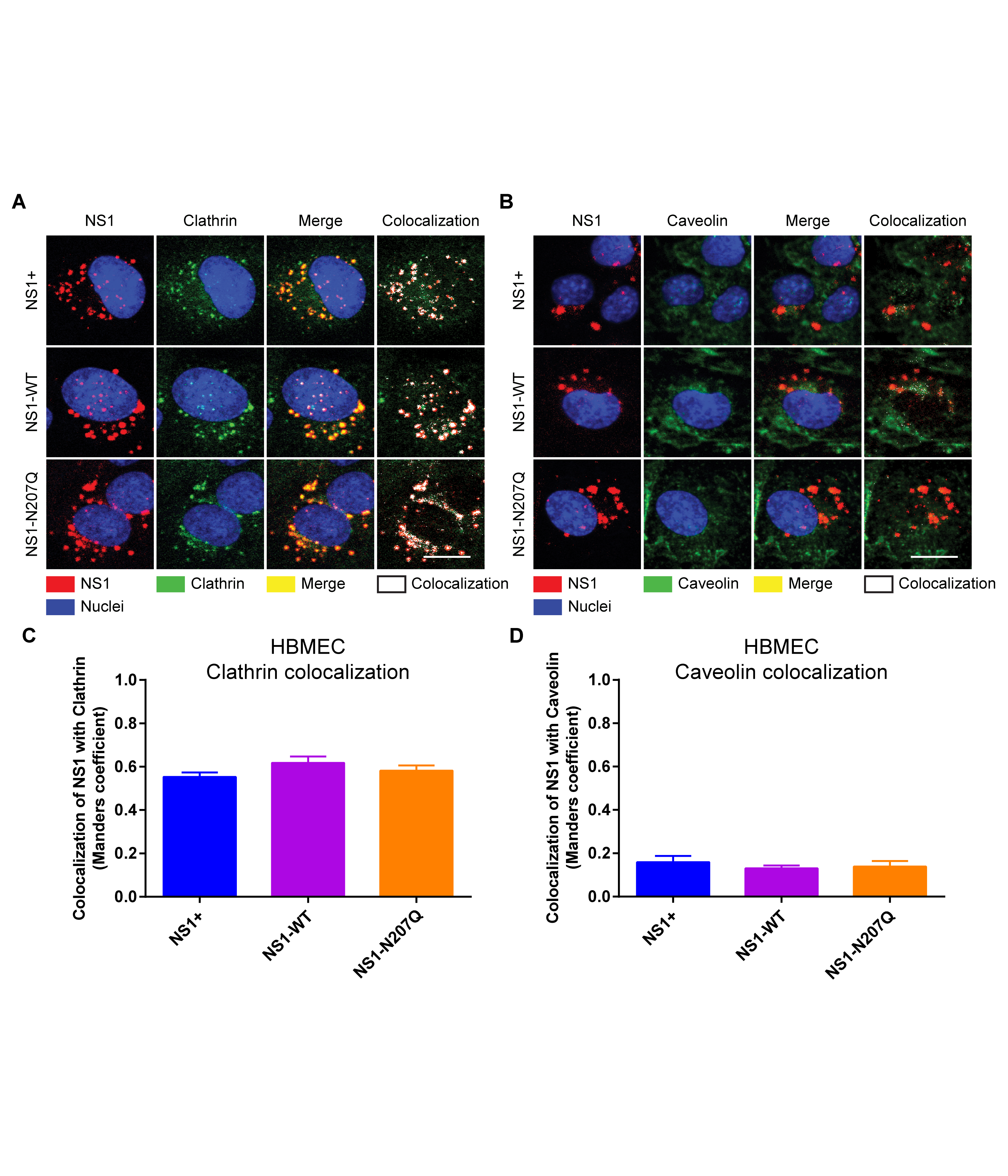

Supplement: S8 Fig — Colocalization of DENV NS1 proteins with either clathrin (A) or caveolin (B) in HBMEC was performed as described in the legend for Fig 4A, except HBMEC were plated instead of HPMEC. Images are 40X; scale bars, 5 μm. Quantification of the amount of spatial overlap between the two signals, NS1 and clathrin (C), or NS1 and caveolin (D) in S8A Fig and S8B Fig, respectively. The means ± SEM of two individual experiments run in duplicate are shown. (TIF) [file ppat.1007938.s009.tif]

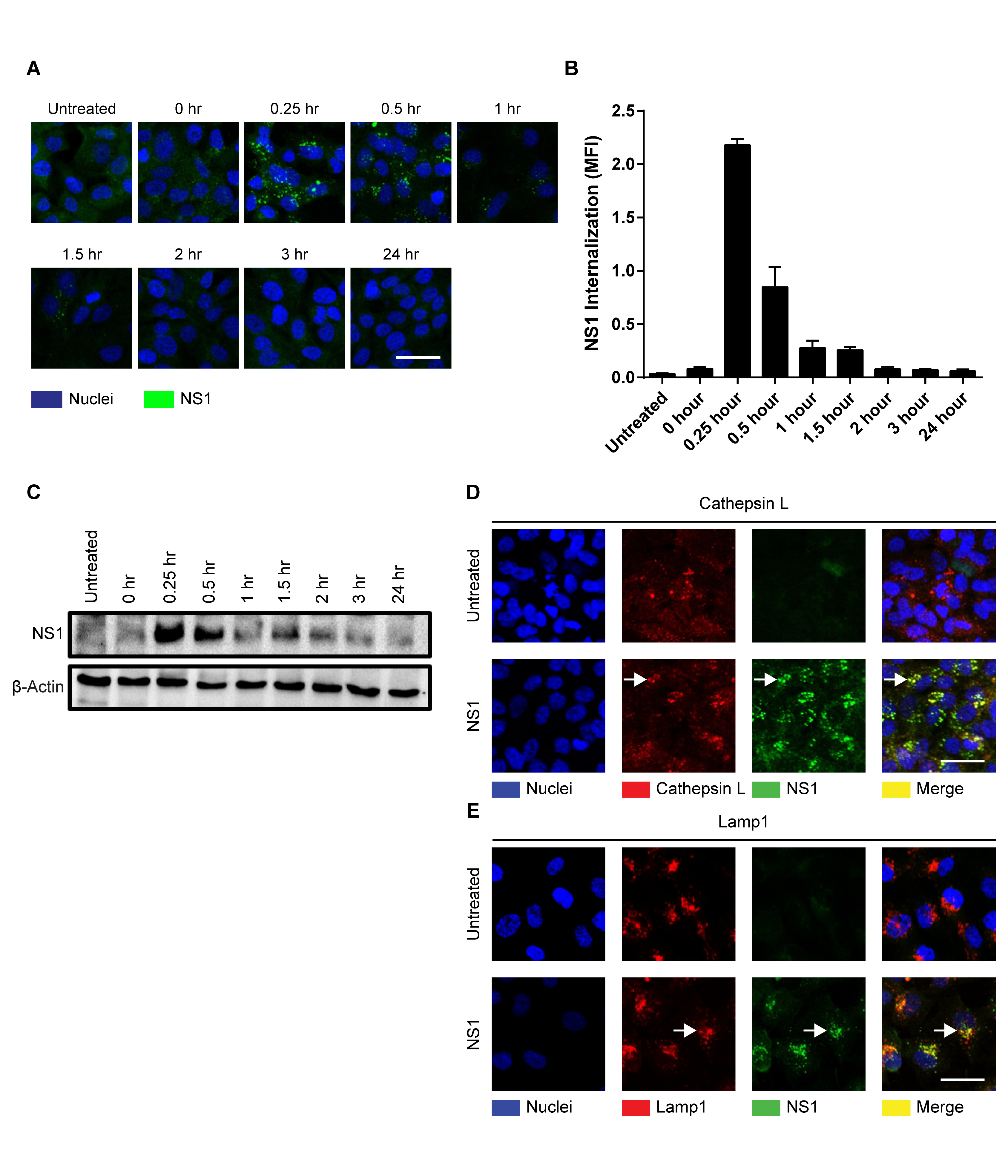

Supplement: S9 Fig — (A) Internalization of NS1 (10 μg/ml) into HPMEC was measured at the indicated time-points after allowing NS1 to adsorb to the cell surface at 4°C. After NS1 adsorption, cells were washed twice in cold PBS to remove unbound NS1 and were moved to 37°C for the indicated times. At the indicated time-points, cells were fixed, permeabilized, and visualized by IFA. Before fixation, cells were washed in a glycine-acid buffer to remove NS1 that is bound to the surface but not yet internalized. NS1 is visualized by an anti-6xHis-tag antibody (green), and nuclei of cells are stained with Hoechst (blue). Images (20X; scale bars, 50 μm) are representative of two independent experiments. (B) MFI quantification of internalized NS1 in A. (C) Same as A, except cells were collected in protein sample buffer for Western blot analysis. Before cell lysis, cells were washed in a glycine-acid buffer to remove NS1 that is bound to the surface but not yet internalized. (D) NS1 was allowed to internalize into HPMEC for 90 minutes (unbound NS1 was not washed away), cells were then fixed and permeabilized, and colocalization of NS1 and cellular markers was visualized by IFA. NS1 was detected with an anti-6xHis-tag antibody (green, the secondary antibody was conjugated to AF488). Lamp1 (red, the secondary antibody was conjugated to AF568); CTSL, cathepsin L (red, the secondary antibody was conjugated to AF647). Images (20X; scale bars, 25 μm) are representative of two independent experiments. (TIF) [file ppat.1007938.s010.tif]

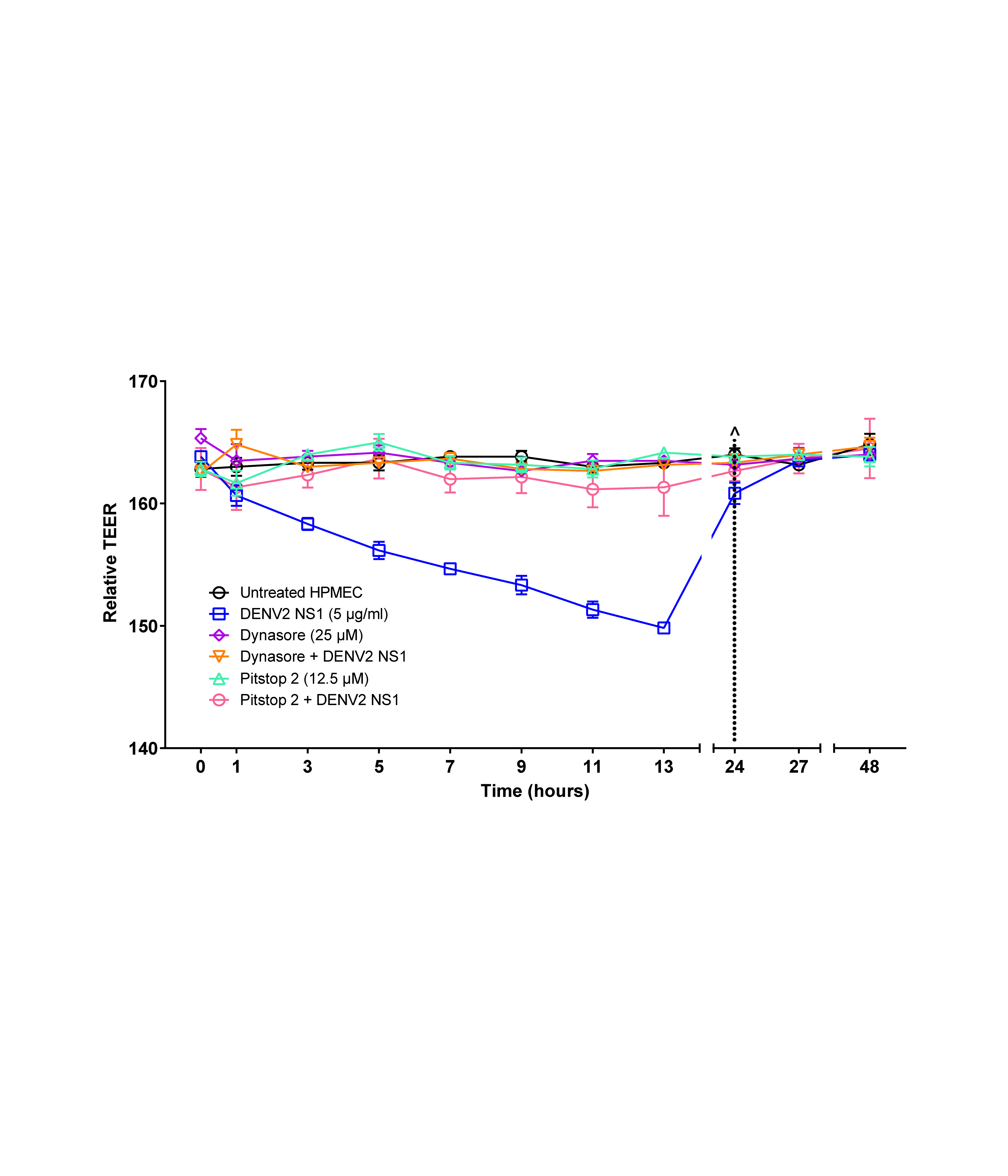

Supplement: S10 Fig — Transendothelial electrical resistance (TEER) assays were used to determine the effect of inhibiting clathrin-mediated endocytosis on NS1-induced hyperpermeability. TEER data here are the non-normalized raw data from Fig 5C displayed in Ohms (Ω). (TIF) [file ppat.1007938.s011.tif]

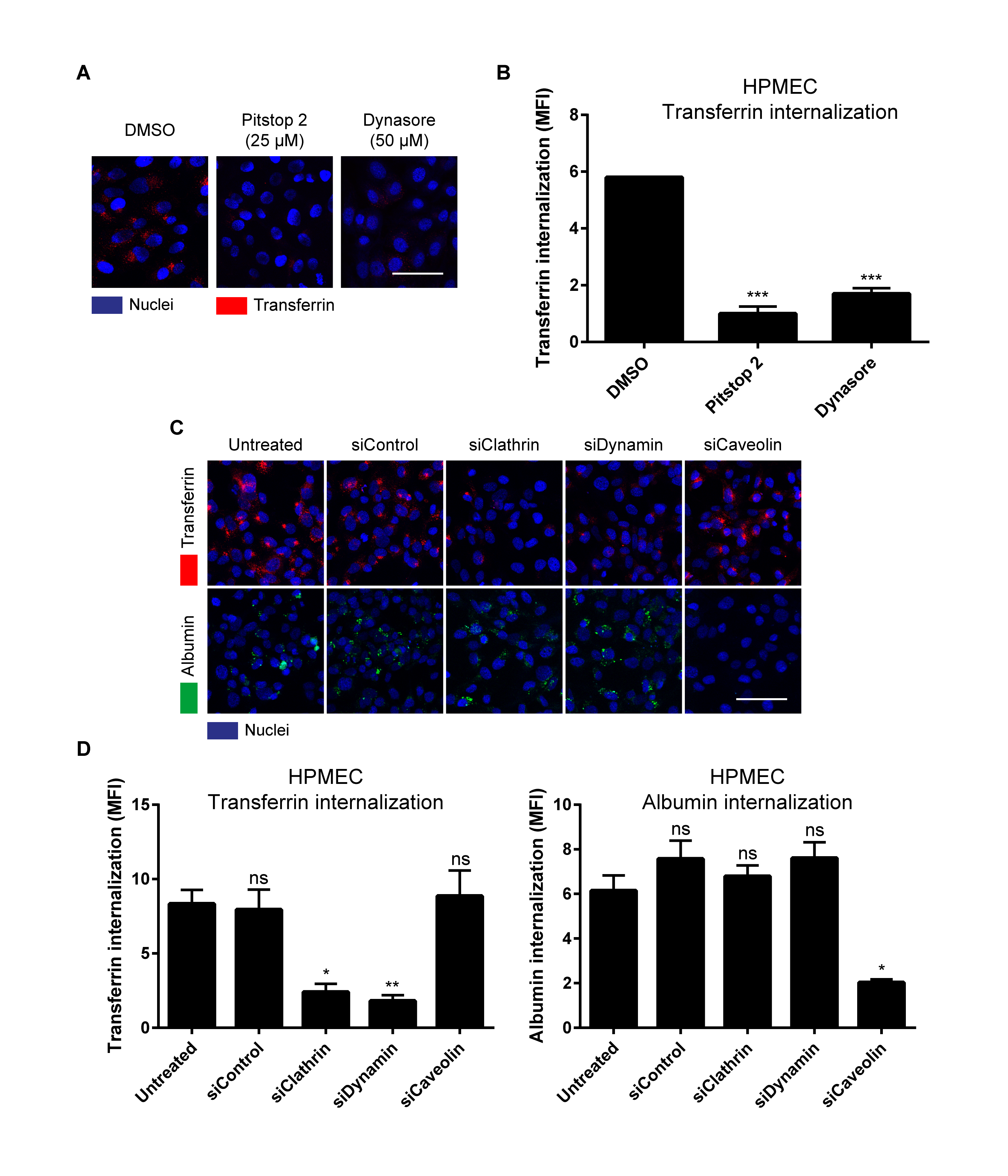

Supplement: S11 Fig — Internalization of (A-D) transferrin-Alexa 568 and (C, D) albumin-FITC in HPMEC monolayers grown on coverslips and (A, B) treated with chemical inhibitors (30 minutes at 37°C; Pitstop 2 = 25 μM; Dynasore = 50 μM) or (C, D) transfected with distinct siRNAs. Internalization of (A-D) transferrin conjugated to Alexa-568 (20 μg/ml), to control for clathrin-mediated endocytosis, and (C, D) albumin-FITC (100 μg/ml), to control for caveolin-mediated endocytosis, was determined after 10 minutes and 1.5 hours at 37°C, respectively. After this time, cell monolayers were washed (4X) with an acid buffer to remove bound-non-internalized proteins. The amount of internalized transferrin and albumin was determined by confocal microscopy imaging (images: 20X; scale bars, 25 μm) and expressed as mean fluorescence intensity (MFI). The means ± SEM of two (B, D right) or three (D left) individual experiments run in duplicate are shown. ns, not significant; *, p<0.05; **, p<0.01. (TIF) [file ppat.1007938.s012.tif]

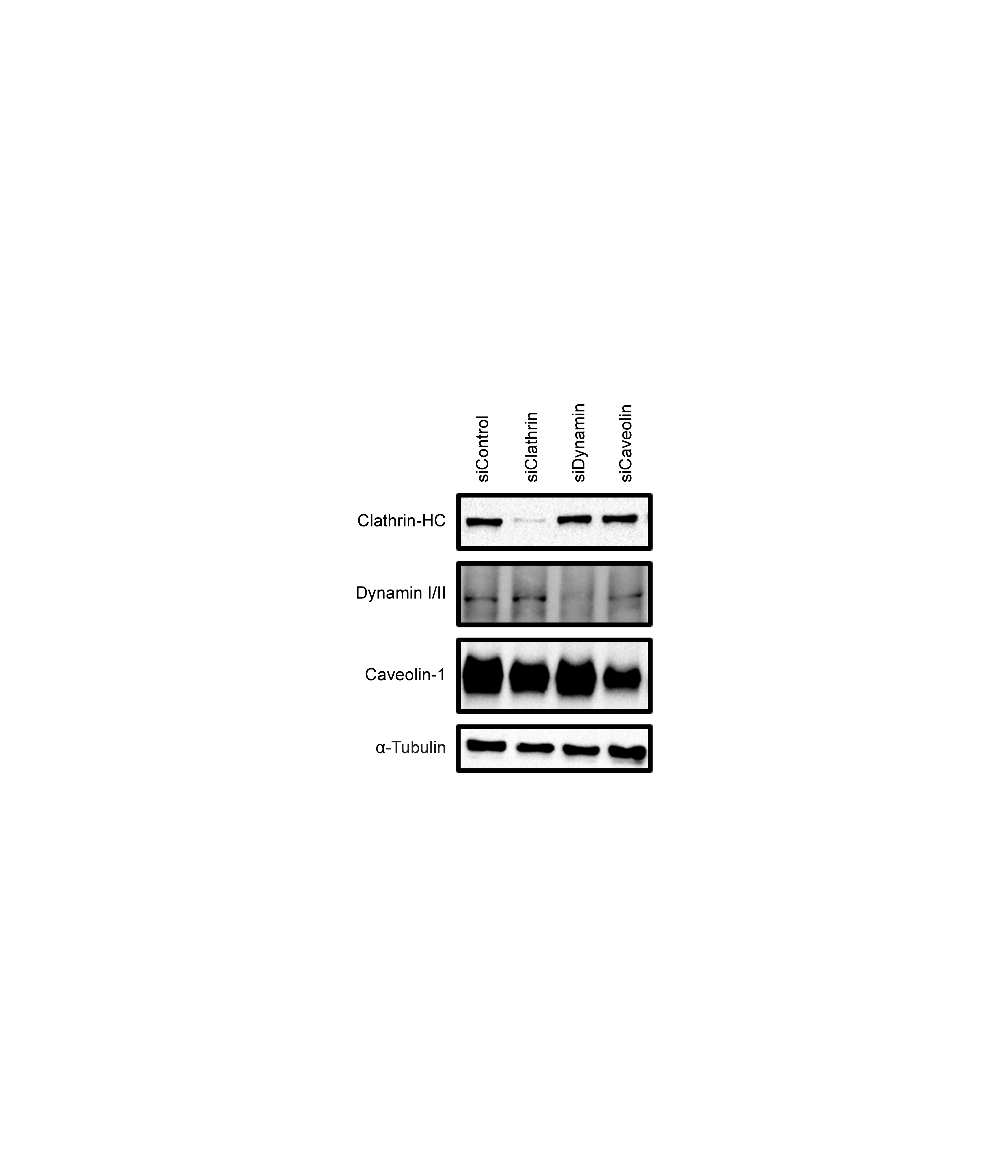

Supplement: S12 Fig — Western blot analysis of cell lysates from Fig 6A and 6B and S11 Fig., to test knock-down efficiency of clathrin heavy chain, dynamin I/II, and caveolin-1. Representative image shown from 3 independent Western blots. α-Tubulin is included as a loading control. (TIF) [file ppat.1007938.s013.tif]

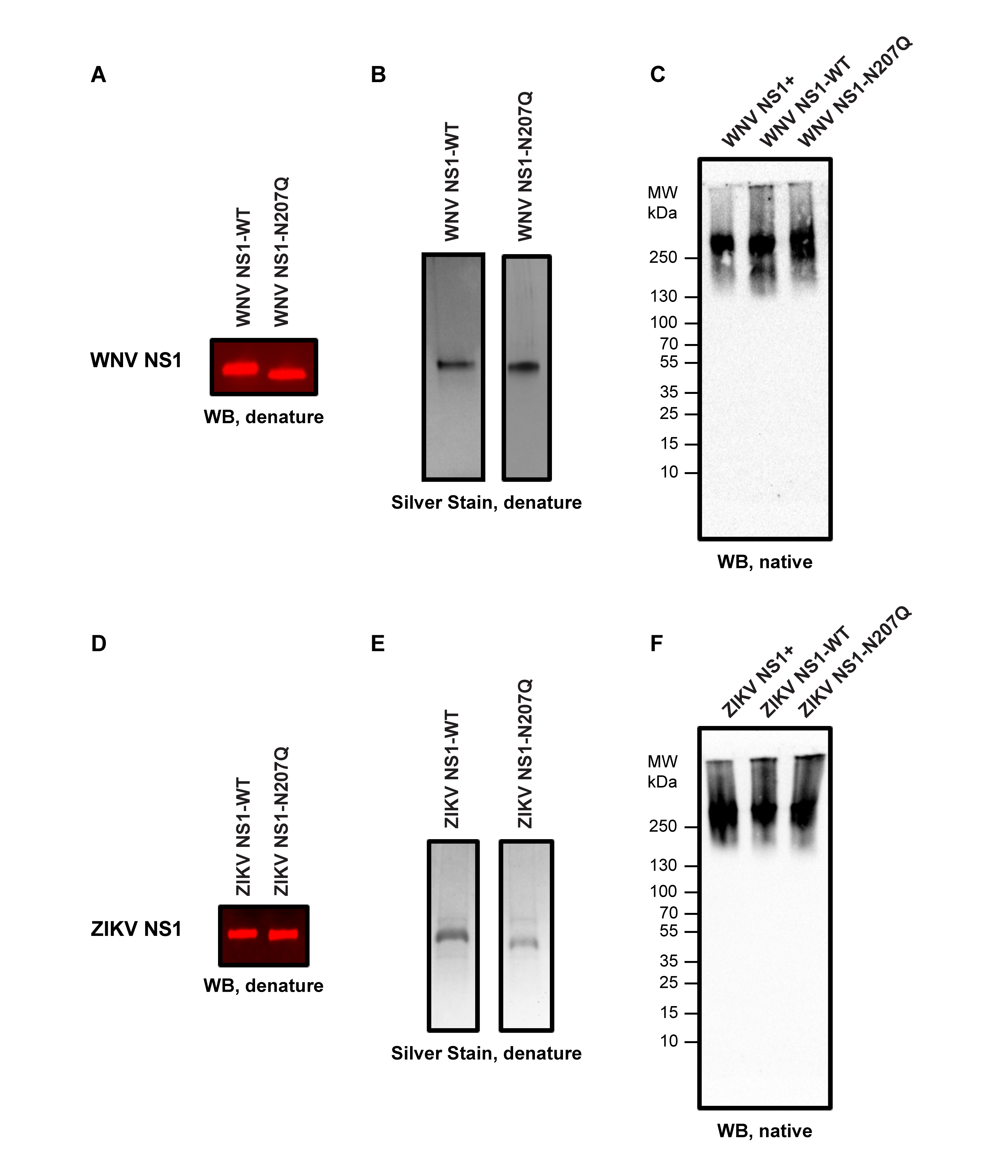

Supplement: S13 Fig — (A and D) Western blot analysis, under denaturing conditions, of supernatants from 293F cells transfected with the indicated construct. (B and E) Silver staining analysis after SDS-PAGE of the indicated purified NS1 proteins. Gels depict NS1 monomers. (C and F) Western blot analysis, in native condition, of the indicated purified NS1 proteins or commercially purchased (NS1+). For all Western blots NS1 was detected using an anti-6xHis-tag antibody. (TIF) [file ppat.1007938.s014.tif]

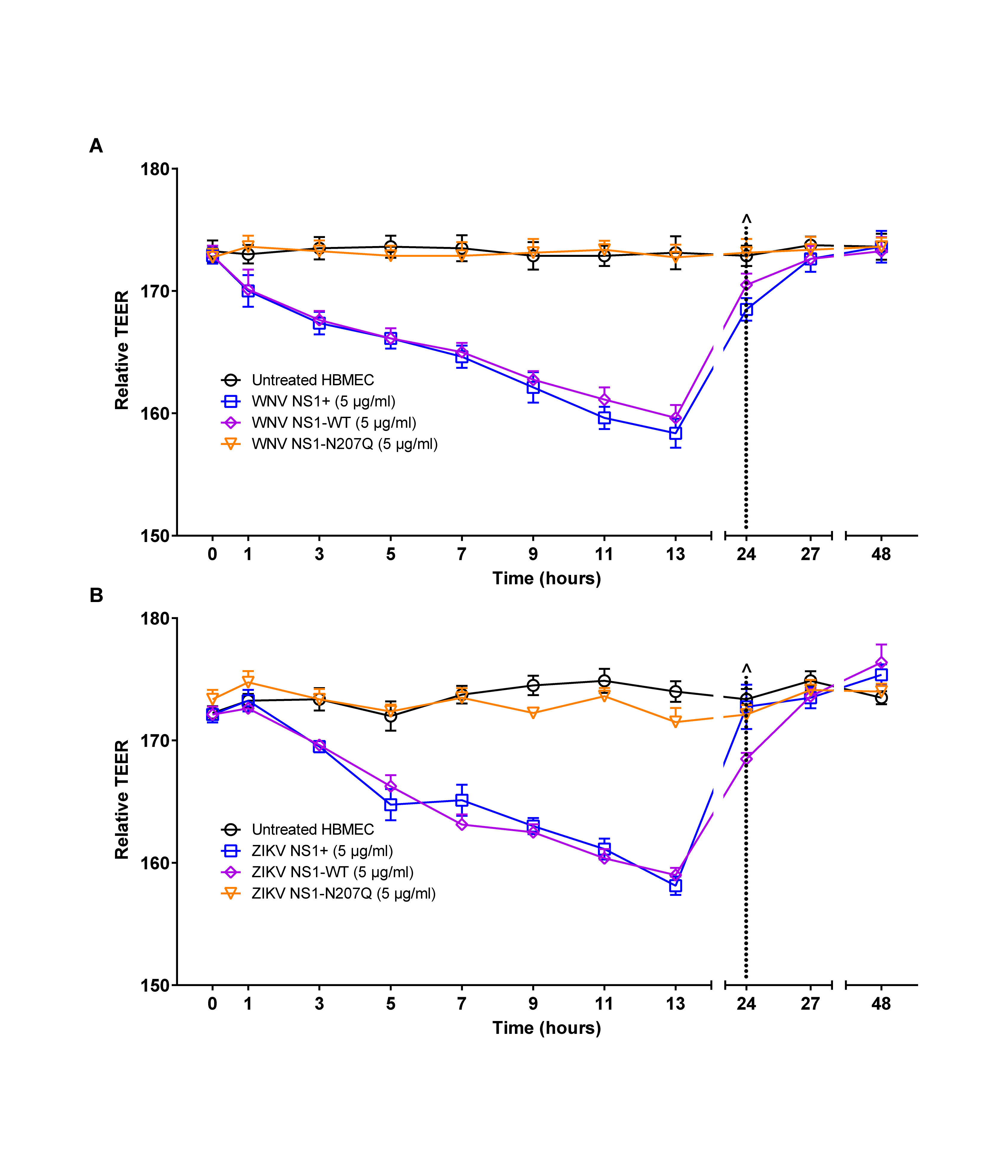

Supplement: S14 Fig — Transendothelial electrical resistance (TEER) assays were used to determine the effect of the WNV and ZIKV NS1-N207Q mutant on NS1-induced hyperpermeability. TEER data here are the non-normalized raw data from Fig 8 displayed in Ohms (Ω). (A) Values from HBMEC treated with WNV NS1 from Fig 8B. (B) Values from HBMEC treated with ZIKV NS1 from Fig 8C. (TIF) [file ppat.1007938.s015.tif]

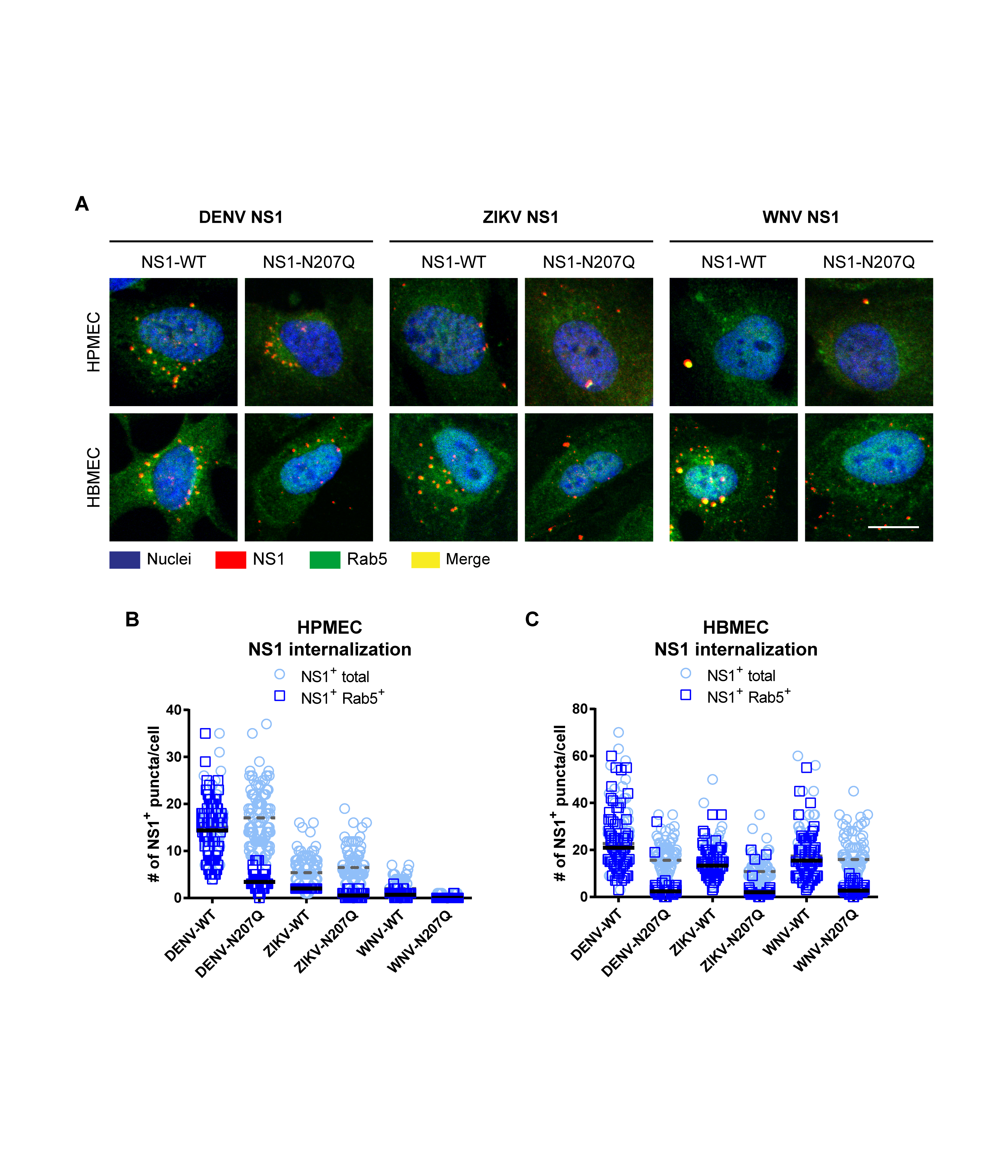

Supplement: S15 Fig — HPMEC (A top, B) and HBMEC (A bottom, C) monolayers grown on coverslips were treated with three different flavivirus NS1 proteins (DENV, ZIKV and WNV: 10 μg/ml) including NS1-WT and the NS1-N207Q mutant. NS1 binding and internalization were examined after 1.5 hours of incubation at 37°C using confocal microscopy to detect the co-staining of NS1 protein (red) and the early endosomal marker Rab5 (green). Nuclei were stained using Hoechst (blue). Images (40X; scale bars, 5 μm) are representative of 2 independent experiments. (B, C) The amount of total NS1 in each endothelial cell (red staining in A) was expressed as NS1+, total puncta per cell (light blue open circles), and NS1 puncta colocalized with Rab5 (yellow staining in A) was expressed as NS1+ Rab5+ puncta, indicating NS1 colocalization and therefore internalization (dark blue open squares). NS1+ puncta were counted from a total of 200 cells collected from two independent experiments and analyzed using ImageJ. Dashed lines (light grey) and solid lines (black) represent the mean values for NS1+ total puncta and NS1+Rab5+ puncta, respectively. (TIF) [file ppat.1007938.s016.tif]
